# Decoding common and complex genotypes of facioscapulohumeral muscular dystrophy using methylation profiling and long-read sequencing

**DOI:** 10.1093/braincomms/fcag370

**Published:** 2026-10-10

**Authors:** Florentine Scharf, Morghan C Lucas, Stefanie Gehling, Felix Kleefeld, Veronika Schönrock, Anna Benet-Pagès, Hans-Werner Rausch, Lucas Schirmer, Jochen Schäfer, Annika Saak, Joseph Porrmann, Sylvia Hütter, Christina Emde, Anne Boblest, Svenja Neuhoff, Tim Hagenacker, Sebastian Burkart, Sebastian Mingers, Ann-Cathrine Berking, Lisa Olfe, Corinna Hendrich, Kristin Schröck, Elizabeta Sauer, Agata A Mossakowski, Kai Sendelbach, Udo Koehler, Kerstin Becker, Tobias Bartolomaeus, Ariane Hallermayr, Barbara Klink, Teresa Neuhann, Maggie C Walter, Benedikt Schoser, Angela Abicht, Hannes Erdmann

**Affiliations:** MGZ - Medizinisch Genetisches Zentrum, Munich 80335, Germany; MGZ - Medizinisch Genetisches Zentrum, Munich 80335, Germany; Friedrich Baur Institute at the Department of Neurology, LMU University Hospital, Ludwig-Maximilians-Universität München, Munich 80336, Germany; Department of Medicine IV, LMU University Hospital, Ludwig-Maximilians-Universität München, Munich 80336, Germany; MGZ - Medizinisch Genetisches Zentrum, Munich 80335, Germany; Department of Neurology, BG University Hospital Bergmannsheil, Ruhr-University Bochum, Bochum 44789, Germany; BG University Hospital Bergmannsheil, Heimer Institute for Muscle Research, Bochum 44789, Germany; Department of Neurology with Experimental Neurology, Charité-Universitätsmedizin Berlin, Corporate Member of Freie Universität Berlin, Humboldt Universität zu Berlin, and Berlin Institute of Health (BIH), Berlin 10117, Germany; MGZ - Medizinisch Genetisches Zentrum, Munich 80335, Germany; MGZ - Medizinisch Genetisches Zentrum, Munich 80335, Germany; Institute of Neurogenomics, Helmholtz Center Munich, Neuherberg 85764, Germany; Department of Neurology and Mannheim Center for Translational Neuroscience, Medical Faculty Mannheim, Heidelberg University, Mannheim 68167, Germany; Department of Neurology and Mannheim Center for Translational Neuroscience, Medical Faculty Mannheim, Heidelberg University, Mannheim 68167, Germany; Department of Neurology, University Hospital Carl Gustav Carus at TUD Dresden University of Technology, Dresden 01307, Germany; Department of Neurology, University Hospital Linz, Linz 4021, Austria; Institute for Clinical Genetics, University Hospital Carl Gustav Carus at TUD Dresden University of Technology, Dresden 01307, Germany; Institute for Clinical Genetics, University Hospital Carl Gustav Carus at TUD Dresden University of Technology, Dresden 01307, Germany; Department of Neuropediatrics, University Hospital Carl Gustav Carus at TUDDresden University of Technology, Dresden 01307, Germany; Department of Neuropediatrics, University Hospital Carl Gustav Carus at TUDDresden University of Technology, Dresden 01307, Germany; Department of Neurology and Center for Translational Neuro- and Behavioral Sciences (C-TNBS), University Hospital Essen, Essen 45147, Germany; Department of Neurology and Center for Translational Neuro- and Behavioral Sciences (C-TNBS), University Hospital Essen, Essen 45147, Germany; Institute of Human Genetics, University Hospital Heidelberg, Heidelberg 69120, Germany; Neurologie Mingers, Übach-Palenberg 52531, Germany; Institute for Human Genetics, Hannover Medical School, Hannover 30625, Germany; Institute for Human Genetics, Hannover Medical School, Hannover 30625, Germany; Institute for Human Genetics, Hannover Medical School, Hannover 30625, Germany; Mitteldeutscher Praxisverbund Humangenetik, Leipzig 04103, Germany; Clinical Genetics and Genomic Medicine, University Hospital Würzburg, Würzburg 97080, Germany; Department of Neurology with Experimental Neurology, Charité-Universitätsmedizin Berlin, Corporate Member of Freie Universität Berlin, Humboldt Universität zu Berlin, and Berlin Institute of Health (BIH), Berlin 10117, Germany; MGZ - Medizinisch Genetisches Zentrum, Munich 80335, Germany; MGZ - Medizinisch Genetisches Zentrum, Munich 80335, Germany; MGZ - Medizinisch Genetisches Zentrum, Munich 80335, Germany; MGZ - Medizinisch Genetisches Zentrum, Munich 80335, Germany; MGZ - Medizinisch Genetisches Zentrum, Munich 80335, Germany; Department of Medicine IV, LMU University Hospital, Ludwig-Maximilians-Universität München, Munich 80336, Germany; MGZ - Medizinisch Genetisches Zentrum, Munich 80335, Germany; MGZ - Medizinisch Genetisches Zentrum, Munich 80335, Germany; Friedrich Baur Institute at the Department of Neurology, LMU University Hospital, Ludwig-Maximilians-Universität München, Munich 80336, Germany; Friedrich Baur Institute at the Department of Neurology, LMU University Hospital, Ludwig-Maximilians-Universität München, Munich 80336, Germany; MGZ - Medizinisch Genetisches Zentrum, Munich 80335, Germany; Friedrich Baur Institute at the Department of Neurology, LMU University Hospital, Ludwig-Maximilians-Universität München, Munich 80336, Germany; MGZ - Medizinisch Genetisches Zentrum, Munich 80335, Germany; Friedrich Baur Institute at the Department of Neurology, LMU University Hospital, Ludwig-Maximilians-Universität München, Munich 80336, Germany

**Keywords:** FSHD, long-read sequencing, methylation, nanopore sequencing

## Abstract

Due to the complexity of the genomic region, the relationship between genotype, phenotype and epigenetics in facioscapulohumeral muscular dystrophy remains incompletely understood, and diagnostics are limited in precision. In this study, we applied Oxford Nanopore Technology long-read sequencing to characterize FSHD1 and FSHD2 patients with common and previously confirmed genotypes (*n* = 14). This approach enabled the simultaneous determination of allele-specific methylation profiles, haplotypes and repeat lengths. Our results confirm the presence of an asymmetric methylation gradient increasing from the proximal to the distal end of 4q35 repeat arrays with a steeper slope in FSHD1 (2.5–8.1%/repeat unit) compared to the two FSHD2 patients (0.3 and 1.2%/repeat unit) studied. The slope is, therefore, a potential marker to differentiate both types of facioscapulohumeral muscular dystrophy, being less biased by repeat length compared to parameters determined in current bisulfite sequencing-based methylation assays. By determining distal methylation allele-specifically, long-read sequencing overcomes a major limitation of such assays. Distal methylation of pathogenic (range 22–45%) and non-pathogenic repeat arrays (range 77–87%) (*P* < 0.0001) is clearly separated and shows a stronger correlation with age-corrected clinical severity (*r* = −0.81, 95% CI: −0.48 to −0.94) compared to studies based on bisulfite sequencing. Overall, this strengthens distal methylation as a superior diagnostic marker of facioscapulohumeral muscular dystrophy disease status. Detailed clinical and genetic characterization of a second cohort (*n* = 20) comprising patients with complex genotypes (*n* = 11) and FSHD2 (*n* = 9) was performed using our bisulfite sequencing-based methylation profile analysis, complementary methods and in three cases, long-read sequencing. Based on these results, we highlight underrecognized patient groups at risk of misdiagnosis when methylation analysis is not part of the diagnostic workup: (i) patients carrying a 4qA166 repeat array; (ii) patients with underrepresented population backgrounds; (iii) facioscapulohumeral muscular dystrophy patients carrying 11 repeat units in the absence of global hypomethylation; and (iv) FSHD2 patients without a detectable causative variant in known epigenetic suppressor genes (*SMCHD1*, *DNMT3B* and *LRIF1*). We hypothesize that unknown epigenetic modifiers explain the penetrance of repeat arrays with 8–10 repeat units in the European and North American populations, the pathogenicity of some repeat arrays with 11 repeat units and the hypomethylation of the entire 4q35 repeat array in some FSHD2 patients without a detected variant in known epigenetic suppressor genes. From our results, recommendations for standard diagnostics are derived. Finally, we hypothesize a preliminary model linking DNA methylation, known genetic parameters and disease severity.

## Teaser

Scharf *et al*. used a variety of methods, including long-read sequencing, to analyse the genomic region causing facioscapulohumeral muscular dystrophy (FSHD). Genetic, epigenetic and phenotype data were collected for various genotypes. This enabled the identification of groups at high risk of misdiagnosis and provided further insights into FSHD’s genetic basis.

## Graphical Abstract

**Graphical Abstract:**
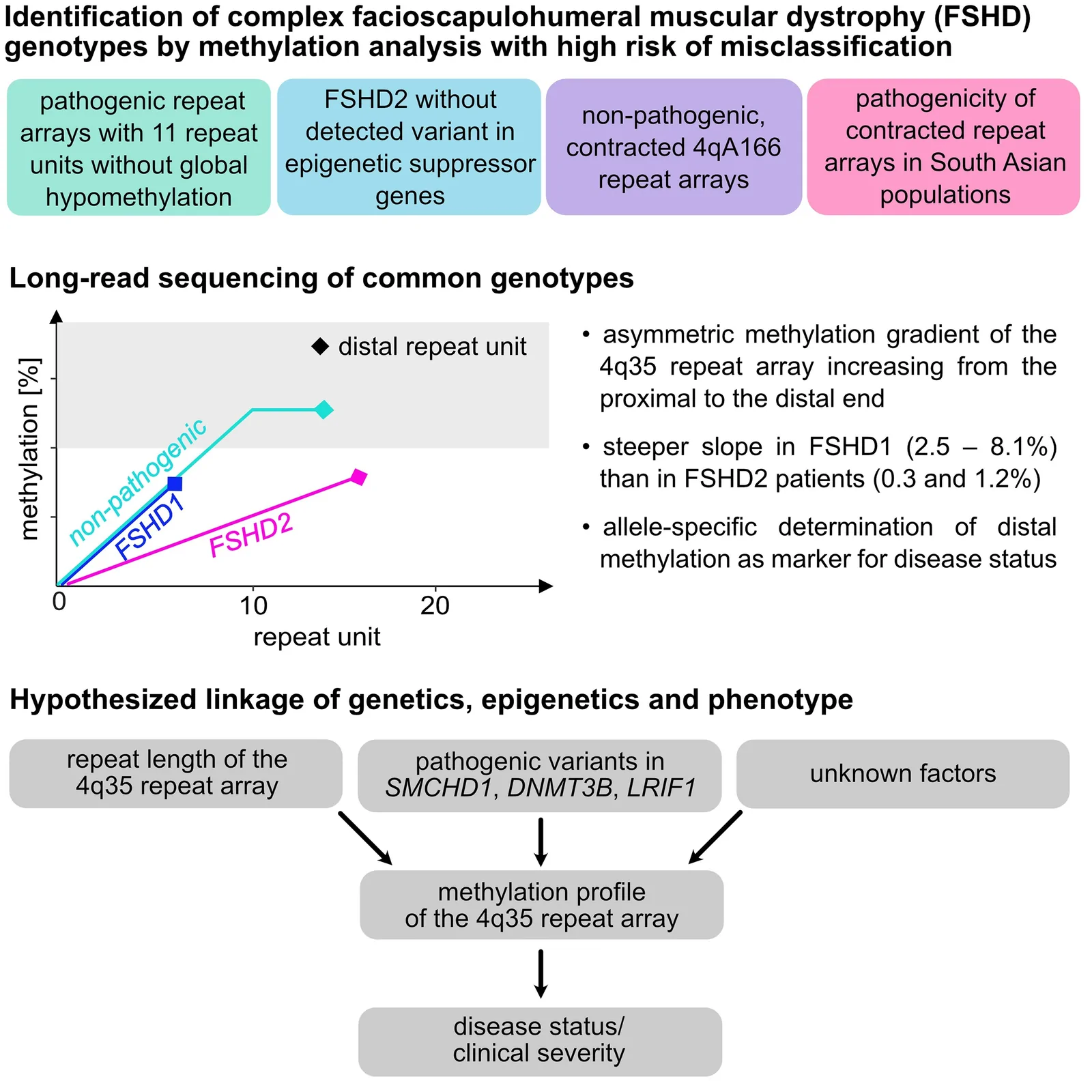

## Introduction

Facioscapulohumeral muscular dystrophy (FSHD, OMIM#158900) is one of the most common muscular dystrophies in Europe.^1^ Based on genetic features, FSHD is categorized into two clinically indistinguishable types: FSHD1 and FSHD2. FSHD1 is linked to contractions of the D4Z4 macrosatellite repeat array on chromosome 4q35, while FSHD2 is associated with globally reduced methylation of the whole repeat array, typically caused by pathogenic variants in the epigenetic suppressor genes *SMCHD1*, *DNMT3B* or *LRIF1*.^2-5^ Both forms require the presence of an intact polyadenylation signal (PAS) encoded by a single nucleotide polymorphism (SNP) within the region flanking the distal repeat unit (RU) that defines the so-called permissive haplotype.^6^

Diagnosis of FSHD typically involves (i) testing for a contracted D4Z4 repeat array (≤10 RU) on a permissive allele to confirm FSHD1 and (ii) testing for pathogenic variants in epigenetic suppressor genes (*SMCHD1*, *DNMT3B* or *LRIF1*) and/or for global hypomethylation in combination with a permissive allele (usually 8–20 RU in length) to confirm FSHD2.^5^ Although diagnostic precision has significantly improved over the last decade, molecular confirmation remains elusive in some patients with a distinct FSHD phenotype, currently referred to as ‘Clinical FSHD, genetic cause unknown’.^5^

The key pathomechanism of both FSHD types is epigenetic derepression of the transcription factor *DUX4*, located within the most distal RU of the 4q35 repeat array and its flanking region. Studies over the last decade confirmed that altered DNA methylation is a molecular correlate of aberrant *DUX4* expression and can serve as a reliable marker of FSHD.^7-12^ Recently, long-read sequencing (LRS) enabled an in-depth characterization of the 4q35 repeat array.^13-15^ By revealing an asymmetric methylation gradient that increases from the proximal to the distal end, these studies provided further insights into the genetic and epigenetic basis of FSHD (Fig. 1A).

**Figure 1:**
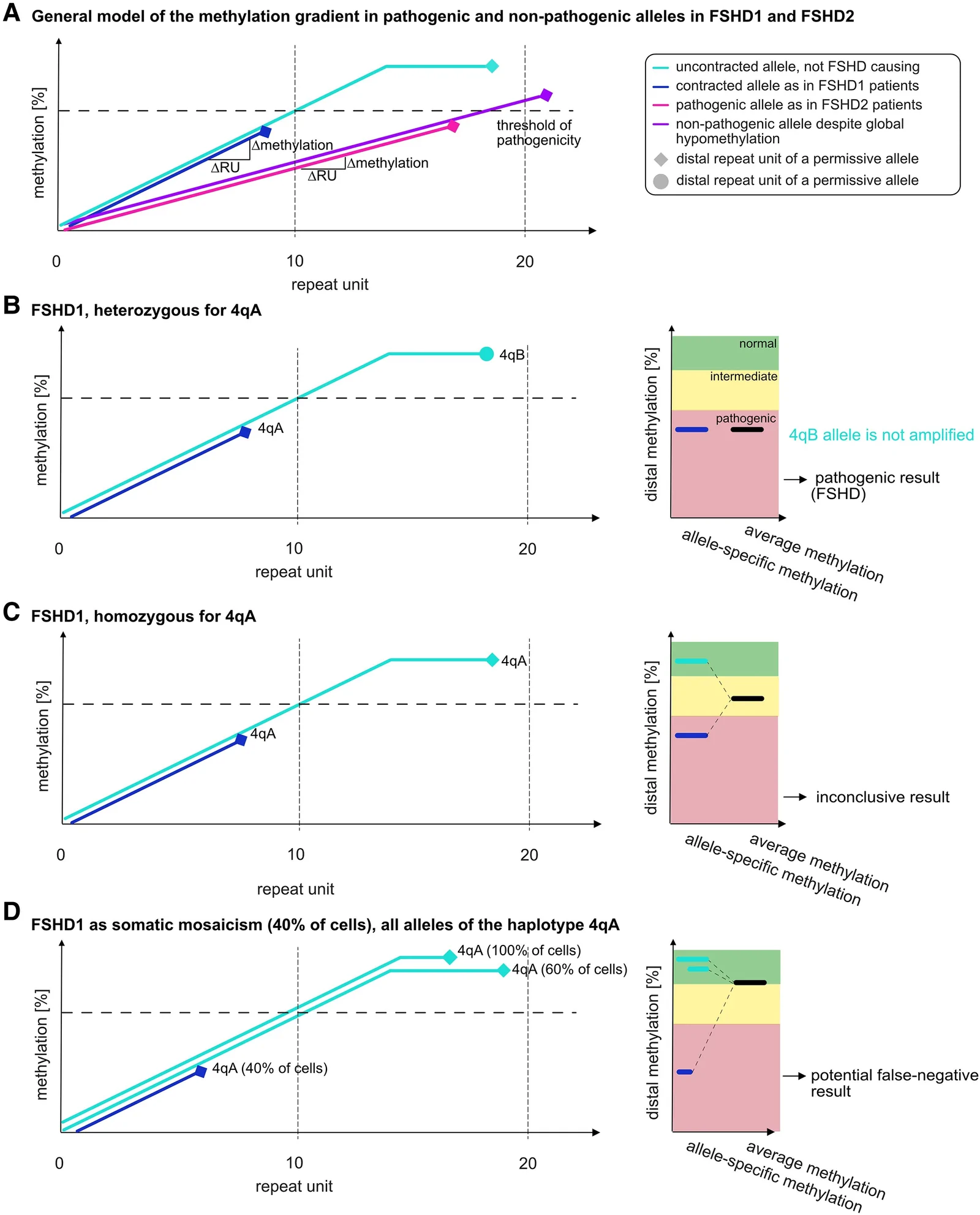
**Illustration of the recently discovered asymmetric methylation gradient of 4q35 repeat arrays and potential biases in determining distal methylation in bisulfite sequencing-based methylation assays.** (**A**) Schematic representation of the increase in methylation from the proximal to the distal end of 4q35 repeat arrays in FSHD1 and FSHD2 patients. In FSHD1 patients, a larger increase in methylation per RU represented by a steeper slope of the methylation gradient (Δmethylation/ΔRU) is observed. Methylation within the most distal RU of a contracted repeat array is below the pathogenicity threshold and therefore causative for FSHD1. Distal methylation of non-pathogenic, uncontracted repeat arrays is above the pathogenicity threshold. In FSHD2 patients, the methylation gradient’s slope is flatter. Repeat arrays causing FSHD2 are usually shorter than 20 RUs and, therefore, have a distal methylation below the pathogenicity threshold. Large repeat arrays can be non-disease-causing, likely associated with a distal methylation above the threshold of pathogenicity. The exact threshold for repeat length in FSHD2 remains to be determined and likely differs inter-individually. (**B**) Average distal methylation (right diagram, right bar) solely reflects the methylation of the contracted permissive repeat array (right diagram, left bar) as the second non-permissive repeat array is not amplified, leading to correct pathogenic results. (**C**) Reduced distal methylation of the disease-causing contracted repeat array (right diagram, lower left bar) is diluted by a highly methylated, non-contracted permissive repeat array on the second allele (right diagram, upper left bar), also carrying the permissive haplotype. Consequently, average distal methylation (right diagram, right bar) is often in the intermediate, inconclusive range. (**D**) Reduced distal methylation of a contracted repeat array present as somatic mosaicism (right diagram, lower left bar) is strongly diluted by permissive repeat arrays present on the other alleles and in different cell populations (right diagram, upper left bars), respectively. In case of a low allele frequency, average distal methylation (right diagram, right bar) is within the normal range, potentially leading to a false-negative result.

Methylation assays are an evolving standalone approach to FSHD testing.^7,9,12,16,17^ Current assays, such as our recently reported FSHD methylation profile analysis (FSHD-MPA), determine two different methylation values of 4q35 repeat arrays: ‘distal’ and ‘global methylation’.^7,12^

‘Distal methylation’, measured within the most distal RU, is a robust marker of overall disease status (Supplementary Fig. 1A and B, Fig 1B and C). It does not allow to differentiate FSHD1 from FSHD2. Distal methylation shows an inverse correlation with age-corrected disease severity and is a potential quantitative biomarker.^7-9,11,12,16-18^ The parameter is usually assessed by bisulfite sequencing (BSS)-based assays utilizing primers specific for permissive 4qA (and 4qAL) repeat arrays. However, these approaches lack allele specificity, particularly limiting sensitivity in patients who are homozygous for the permissive haplotype or who carry somatic mosaicism (Fig. 1C and D). In these cases, distal hypomethylation from the pathogenic repeat array on one allele is diluted by highly methylated, non-pathogenic repeat arrays on the other allele, potentially leading to inconclusive or, in isolated cases, false-negative results.

‘Global methylation’, measured as the average methylation across all 4q35 and 10q26 repeat arrays, is used to differentiate FSHD1 and FSHD2 (Supplementary Fig. 2A and B). The parameter is determined by assaying a region present in each RU of 4q35 and 10q26 repeat arrays (DUX4 in FSHD-MPA and DR1 in other assays, Supplementary Fig. 1A and C).^19^ In FSHD2, global methylation is reduced compared to FSHD1 and non-affected individuals. Global methylation levels are biased by repeat length, as shown by a moderate correlation between DR1 methylation level and the combined lengths of the 4q35 and 10q26 repeat arrays in a recent study.^20^ As such, large repeat arrays can artificially increase global methylation (Supplementary Fig. 2D). This potentially leads to a global methylation in the intermediate range in some FSHD2 patients and, therefore, inconclusive results. Conversely, additional short repeat arrays may cause artificially low global methylation levels within the pathogenic or intermediate range in some FSHD1 cases (Supplementary Fig. 2C), mimicking FSHD2.

In this study, we used Oxford Nanopore Technology (ONT) LRS to characterize the methylation profile of known FSHD genotypes (Cohort 1), confirm previous findings of an asymmetric methylation gradient and highlight methylation as a superior diagnostic marker.^13-15,21^ In a second cohort (Cohort 2), we describe complex genotypes that were primarily characterized using our BSS-based FSHD-MPA method and, in three cases, using ONT LRS. These cases highlight patient groups with a high risk of being misclassified and yield insights into the epigenetic basis of FSHD. We summarize the results of both cohorts by presenting a model for the relationship between genetics, epigenetics and phenotype in FSHD and provide recommendations for the diagnostics of FSHD.

## Materials and methods

### Patients and study approval

A total of 34 individuals previously analysed in our institute, divided into two cohorts, were analysed in this study. Cohort 1 (Supplementary Tables 1 and 2) included 14 patients with known genotypes and previously confirmed diagnoses of FSHD1 (12 patients, A1–A12), FSHD2 based on a pathogenic variant in *SMCHD1* (one patient, B1, referred to as ‘FSHD2*_SMCHD1_*’) and FSHD1+2 with unknown origin of hypomethylation (one patient, C1, referred to as ‘FSHD1+2_no variant’_), all analysed using ONT LRS to implement the method. Methods used to establish the initial diagnosis varied depending on the time point of analysis and the material available. Cohort 2 comprised 20 patients (Supplementary Tables 1–4) with either complex genotypes (11 patients, D1, E1–6, F1 and G1–3) or FSHD2 (9 patients, B2–B10). For a comprehensive characterization, all patients were analysed using established diagnostic methods as summarized in Supplementary Table 1. For FSHD2 patients without a variant in known epigenetic suppressor genes (referred to as ‘FSHD2_no variant_’), multiplex ligation probe amplification (MLPA) of *SMCHD1* and NGS analysis of FSHD2 candidate genes (*CTCF*, *DNMT1*, *DNMT3A*, *EZH2* and *SUV39H1*) identified in a previous study were performed.^22^ Additionally, Optical Genome Mapping (OGM) of *SMCHD1*, *DNMT3B* and *LRIF1* was performed for five of six patients with appropriate material available. Exome sequencing was performed in all patients of Cohort 2 to identify potential dual diagnoses and rule out alternative ones. ONT LRS was performed for three patients (E2, F1 and G1) of this cohort. Additional patients could not be analysed as ONT R9 flow cells were discontinued during our study and replaced by R10 flow cells, which are largely incompatible with CRISPR/Cas9 target enrichment. Additionally, required amounts of high-molecular-weight DNA were not available for all patients of Cohort 2.

Board-certified neurologists assessed phenotypes. Age-corrected clinical severity (ACSS) was determined as previously described (ACSS = 2000 × CSS / age at examination).^23,24^ Informed consent was obtained from all participants. All genetic analyses and investigations were performed in accordance with the guidelines of the Declaration of Helsinki and approved by the local ethics committee of the Ludwig-Maximilians-University of Munich (vote no. 24-0789).

### Haplotype analysis and methylation profile analysis (FSHD-MPA)

Haplotype analysis following the Tsumagari and KASP assays was performed as previously described.^12,25^ Methylation profile analysis (FSHD-MPA) was performed as previously described with updated cut-off values (Supplementary Table 5).^12^ Pathogenic thresholds were defined as the highest value in FSHD1 or FSHD2 patients that did not overlap with values of negative cases and healthy controls. Upper thresholds for the intermediate range were defined as the lowest value in FSHD1- or FSHD2-negative patients that did not overlap with those of positive cases, as previously established.^16^

Patients were diagnosed as FSHD-positive when distal methylation fell within the pathogenic range. FSHD2 diagnosis was based on an additional global methylation within the pathogenic range. In cases of intermediate distal or global methylation, confirmation or exclusion of FSHD was determined by additional genetic analyses, including repeat length (FSHD1) and pathogenic variants in FSHD2-associated genes (FSHD2).

### OGM

Bionano OGM was performed as previously described to determine D4Z4 repeat lengths and haplotype and to test for structural variants (SVs) and copy number variants (CNVs) in *SMCHD1*, *DNMT3B* and *LRIF1* and in FSHD2 candidate genes (*CTCF*, *DNMT1*, *DNMT3A*, *EZH2* and *SUV39H1*).^22,26-28^ Repeat lengths of contracted repeat arrays (<48 kb) determined by OGM show high concordance with Southern blotting, with a variance of ±1 RU in individual cases, in line with previous studies.^29^

### Southern blotting

Southern blotting was performed as previously described.^12^ The accuracy of the repeat length was determined as ±1 RU for repeat arrays <48 kb.

### Next-generation sequencing (NGS)

Sequencing of *SMCHD1*, *DNMT3B*, *LRIF1* and FSHD2 candidate genes (*CTCF*, *DNMT1*, *DNMT3A*, *EZH2* and *SUV39H1*) as well as exome sequencing was performed as previously described.^12^ Variants were classified according to the ACMG/AMP (American College of Medical Genetics and Genomics/Association for Molecular Pathology) guidelines.^30^

### *SMCHD1*-MLPA

FSHD2 patients identified by FSHD-MPA with negative results of NGS sequencing for variants in *SMCHD1*, *DNMT3B* and *LRIF1* were analysed by MLPA to test for CNVs in *SMCHD1* or to confirm CNVs detected by NGS. Genomic DNA was analysed with the SALSA MLPA P493 SMCHD1 kit (MRC-Holland) according to the manufacturer’s instructions. MLPA products were separated by capillary electrophoresis using the ABI-3130XL Genetic Analyzer (Applied Biosystems, Foster City, CA). Data were analysed with GeneMarker software (Softgenetics, State College, CA), using the MLPA ratio for analysis and peak area for quantification.

### ONT LRS

DNA extraction, library preparation and flow cell loading were performed according to the ONT Cas9-targeted sequencing protocol using ONT’s SQK-CS9109 kit and 5 µg of input gDNA. CRISPR RNAs (crRNAs) to enrich the 4q35 repeat array were designed using CHOPCHOP 8 (Supplementary Table 6, Supplementary Fig. 3). Sequencing was performed with ONT FLO-MIN106D R9 flow cells and with ONT FLO-MIN114 R10 flow cells on the GridION X5 sequencer. Base and methylation calling were performed using guppy v.7.4.12+0e5e75c49 using the model dna_r9.4.1_450bps_modbases_5mc_cg_sup.cfg for R9 flow cells and dna_r10.4.1_e8.2_400bps_5khz_modbases_5hmc_5mc_cg_sup.cfg for R10 flow cells.^27,28^ The generated FASTQ files were aligned to the human reference genome (T2T CHM13-v2.0/hs1) using Minimap2 v.2.27-r1193 to pre-filter for reads potentially spanning the region of interest (chr4:193432996–193543640 and chr10:134604599–134733754).^29^ Of those, reads were further characterized based on specific sequence motifs using blastn v2.11.+. First, reads covering the distal end of the repeat arrays were selected based on the presence of the distal anchor sequence (Supplementary Table 7). These reads were further analysed for the presence or absence of the restriction sites for *XapI* and *BlnI* to distinguish reads originating from chromosome 4q35 (B^−^X^+^) from reads originating from chromosome 10q26 (B^+^X^−^). Repeat length was determined based on the number of *XapI* restriction sites in 4q35 reads containing the distal and proximal anchor sequences (full-length reads) confirmed by manual inspection of mapped reads. Haplotyping was performed based on the absence of the PAS in 4qB and its presence in the cases of 4qA and 4qAL. 4qAL was distinguished based on an inserted sequence in intron 2 of *DUX4* as opposed to 4qA. In case of heterozygosity, single reads were assigned to one of the two 4q35 repeat arrays based on the distinct haplotype. In cases of homozygosity, all full-length reads corresponding to the shorter repeat array were assigned to this allele. All reads longer than this repeat array—regardless of whether they are full-length or partial—were assigned to the second allele. Distal methylation was calculated as the average methylation in chr4:193539784–193543078 using modbam2bed v0.9.5 over the mapped reads, separated by allele; accordingly, proximal methylation was calculated in the most proximal RU completely covered by full-length reads assigned to one allele, with the repeat unit being flanked by *KpnI* restriction sites. Quality control was done using NanoPlot v1.41.6.^30^ Repeat arrays were included in the analysis if supported by a minimum of three allele-specific reads (see the ‘Statistical analysis’ section and Supplementary Fig. 4). For distal methylation, all allele-assigned reads were considered; for the methylation gradient’s slope, reads were additionally restricted to full-length reads. Sequencing depth and quality parameters are given in Supplementary Tables 8 and 9.

### Statistical analysis

Statistical analyses were performed using R v.4.0.0. The significance of differences between two groups was assessed using Welch’s *t*-test (two-tailed). A *P* value < 0.05 was considered statistically significant. Correlation analyses were performed using Pearson’s correlation test, 95% confidence intervals of the correlation coefficients were determined, and *P* values were calculated to test the significance of the correlation. Thresholds of distal methylation defining patients as positive or negative for FSHD were determined as average distal methylation ±2× standard deviation of all disease- and non-disease-causing repeat arrays in Cohort 1, respectively. Linear regression analysis was performed to determine the qualitative relationship between average methylation per RU and repeat length. The slope of the resulting function equals the slope of the methylation gradient. To estimate the minimum number of reads required for reliable calculation of distal methylation and the slope of the methylation gradient, downsampling experiments were performed on full-length reads of the contracted repeat arrays of Cohort 1 for all samples with more than 50 reads on the contracted repeat array, including only one representative of the familial cases A8–A11 (*n* = 9). For each downsampling level (1–10, 15 and 20 reads per allele), 30 replicates were generated by random sampling using Python’s random.sample() function (python3.9), and the interquartile range (IQR) of both distal methylation and methylation gradient’s slope was calculated as a measure of dispersion, and an exponential decay function was fitted to the data. The resulting model confirmed a rapid decay in dispersion at low read depths, with the curve approaching saturation from approximately seven reads per allele onwards. Alleles with fewer than three reads showed substantially elevated dispersion and are not recommended for analysis and excluded from this study. Notably, sample A11 showed substantially higher variability in slope estimates across downsampling replicates, as a linear gradient is more susceptible to noise with fewer data points (2 RU). A conservative minimum threshold of 15 reads per allele is recommended for slope analysis for very short repeat arrays, based on visual inspection of dispersion stabilization (Supplementary Fig. 4, Supplementary Code 1).

## Results

### ONT LRS enables in-depth analysis of the 4q35 repeat array in common FSHD genotypes (Cohort 1)

To enable allele-specific analysis of DNA methylation in FSHD patients, we implemented CRISPR/Cas9-targeted ONT LRS targeting the *p13E11* (proximal) and *pLAM* (distal) regions flanking the D4Z4 repeat array (Supplementary Fig. 3). This approach allows parallel assessment of key diagnostic parameters, including repeat length, haplotype and methylation across the entire 4q35 repeat array. We applied this method to 14 patients with known FSHD genotypes (Cohort 1, Supplementary Tables 1 and 2).

Repeat lengths and haplotypes for the shorter repeat array were consistent with those determined by complementary methods, except for patient C1 (4qAL repeat array), where a discrepancy of one RU was noted between OGM and LRS, being within the known error margin of OGM. A proximal-to-distal methylation gradient was consistently observed across all patients. Contracted repeat arrays showed distal hypomethylation (Fig. 2A and B). Average methylation within the most proximal RU of contracted repeat arrays in FSHD1 patients (A1–A12) was 19% ± 3.3% (range 10–24%), increasing by an average slope of 4.8% ± 1.8% per RU (range 2.5–8.1%/RU), reaching average methylation of 33% ± 7.4% (range 22–45%) within the most distal RU, depending on repeat length (Fig. 2B and C, Supplementary Fig. 5, Supplementary Table 2). Due to insufficient full-length coverage, analysis of uncontracted repeat arrays was restricted to the distal end, where average distal methylation (81% ± 3.5%, range 77–87%) was significantly higher compared to contracted repeat arrays (*P* < 0.0001) without any overlap between both groups.

**Figure 2:**
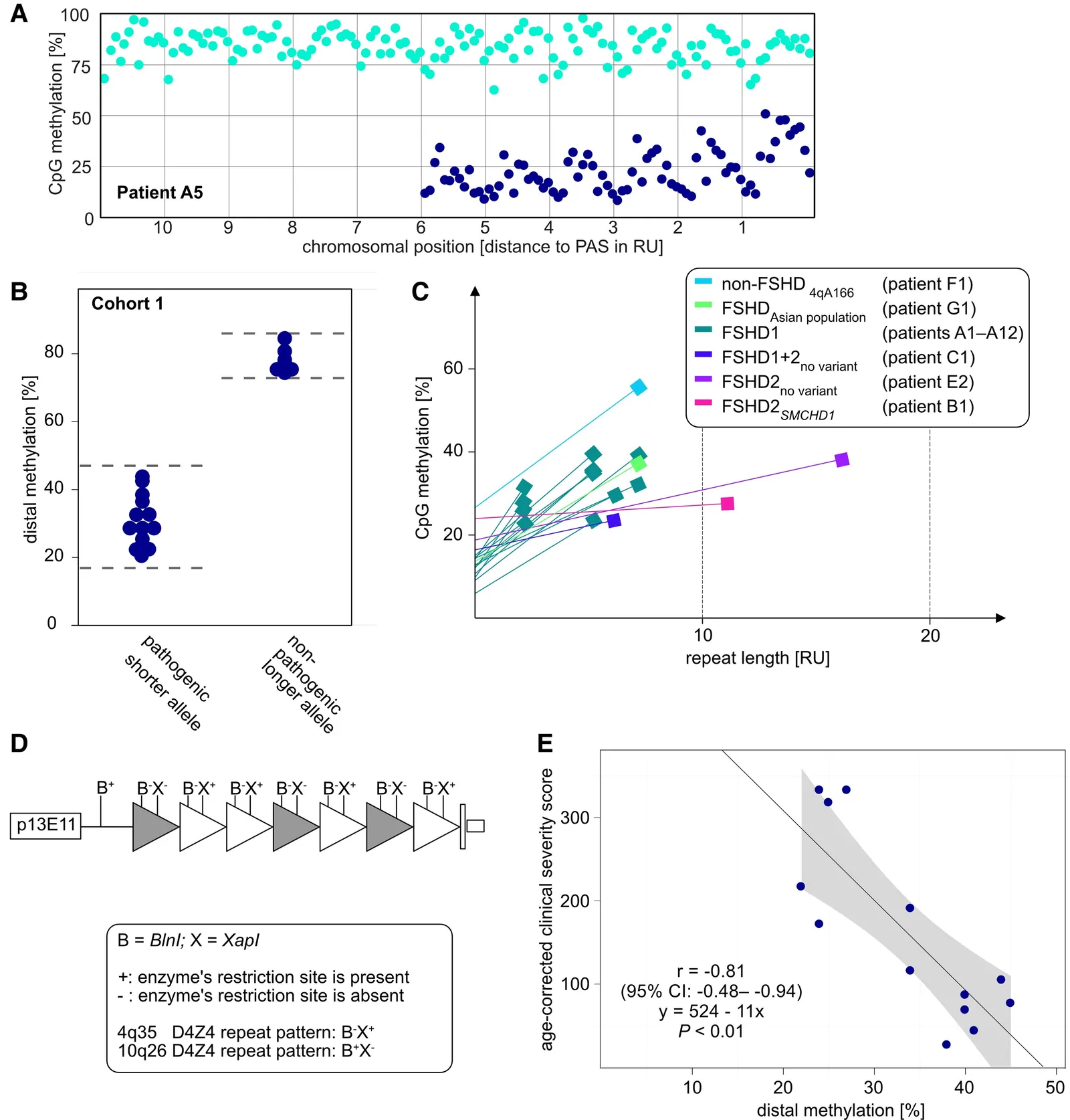
**Results of ONT LRS of patients of Cohorts 1 and 2.** (**A**) Methylation profile of both 4q35 repeat arrays of patient A5 carrying one contracted permissive repeat array with 6 RUs (dots in the bottom) and an uncontracted non-pathogenic repeat array (dots in the top). One dot represents average CpG methylation within a sliding window of 250 base pairs over all reads at respective chromosomal positions relative to the polyadenylation signal within the most distal RU. (**B**) Average methylation of all CpGs within the most distal RU determined over all reads from ONT LRS of all patients of Cohort 1. Left, methylation within the shorter and pathogenic repeat array (*n* = 14, patients A1–12, B1 and C1); right, non-pathogenic and longer repeat array, if covered in the analysis (*n* = 7). As the pathogenicity of the longer repeat array of patient C1 is unknown, it was not included. Dashed lines represent average methylation over all samples ± 2× standard deviation, defined as interval for FSHD-positive (left, 18–48%) and FSHD-negative (right, 74–88%) repeat arrays. (**C**) Course of CpG methylation over the whole repeat array for all patients of Cohort 1 and patients F1, G1, and E2 from Cohort 2 (*n* = 17), determined by regression analysis from average CpG methylation per RU over all reads of the shorter allele and repeat length. While the slope of the methylation gradient is steep in FSHD1 patients (range 2.5–8.1%) and for the complex 4qA166 repeat array (4.0%), it is flatter in the FSHD2*_SMCHD1_* (0.3%), FSHD2_no variant_ (1.2%) and FSHD1+2_no variant_ (1.2%) patients. In the patient with South Asian population background (patient G1), it is within the range of FSHD1 patients (3.3%). (**D**) Composition of the complex 4qA166 repeat array in patient F1 characterized by ONT LRS and in agreement with Southern blotting. Structure was determined based on the presence or absence of restriction sites for the enzymes *BlnI* and *XapI*. The 4qA166 haplotype is currently not associated with FSHD. (**E**) Correlation analysis of allele-specific distal methylation of the shorter, FSHD-causing repeat array with the ACSS for patients of Cohort 1 and 2 (*n* = 13), if detailed clinical data were available. Respective Pearson’s correlation coefficients, their 95% confidence intervals and *P* values are given. Areas indicate the 95% confidence interval of the regression.

In FSHD2*_SMCHD1_* (B1) and FSHD1+2_no variant_ (C1) patients, the shorter permissive repeat array showed distal hypomethylation of 30 and 24%, with corresponding methylation gradients of 0.3%/RU and 1.2%/RU, respectively. For patient C1, the larger 31 RU 4qA repeat array exhibited an approximately linear increase in methylation across the proximal 10 RU, followed by oscillation between 46 and 59% (Supplementary Fig. 5O). The larger repeat array in patient B1 was not sufficiently covered for analysis. Intervals defining patients as positive (18–48%) or negative (74–88%) for FSHD were determined as average distal methylation ± 2× standard deviation of disease- and non-disease-causing repeat arrays in Cohort 1. As the pathogenicity of the longer repeat array in patient C1 is unknown, it was omitted from the calculation.

### Genetic findings in complex and high-risk of being misclassified FSHD genotypes (Cohort 2)

To characterize FSHD genotypes at risk of misclassification, we analysed 20 patients with rare or complex genetic characteristics (Cohort 2) using established diagnostic methods, including FSHD-MPA and ONT LRS for three cases. We identified four key subgroups prone to diagnostic challenges:

#### Exclusion of FSHD in a patient with a complex 4qA166 repeat array

In patient F1 presenting with a severe neuromuscular phenotype, exclusion of FSHD was only possible through haplotype analysis, including ONT LRS. Patient F1 first presented at age 52 with myalgia and elevated creatine kinase levels (3–5× above normal) without muscular weakness and normal EMG and muscle MRI results of the lower limb (Supplementary Table 3). Due to the patient’s Sjögren’s syndrome, overlap myositis was suspected as the underlying cause of the symptoms. Despite immunosuppressive therapy, symptoms rapidly progressed to global respiratory insufficiency, proximal weakness in the upper and lower limbs and axial weakness within 3 years. Muscle biopsy indicated signs of chronic myositis, EMG revealed myopathic changes, and muscle MRI showed signs of myositis and fatty degeneration in the limb-girdle muscles. FSHD was considered as a differential diagnosis. Southern blotting detected a complex repeat contraction (7 RU starting with a non-4q35 RU, Supplementary Table 4). OGM confirmed a contraction of 7 RU and mapped it to chromosome 4q35 *in cis* to the 4qA haplotype without identifying the complex composition of the repeat array. For its further characterization, ONT LRS was performed. The analysis confirmed a contracted repeat array of 7 RU *in cis* to a PAS defining the permissive haplotype (Fig. 2D). Four RUs showed the signature of chromosome 4q [absence of the restriction site of *BlnI* and presence of the restriction site of *XapI* (B^−^X^+^), Supplementary Fig. 6], while three, including the first RU, lacked chromosome 4 and 10 signatures due to resistance to both *XapI* and *BlnI* (B^−^X^−^). Based on a proximal B^−^X^−^ RU, the updated diagnostic guidelines classify the haplotype of the 7 RU repeat array as 4qA166. The haplotype is currently not associated with FSHD despite the presence of a PAS.^5,31^ Therefore, the patient was diagnosed as negative for FSHD. We additionally determined the haplotype based on SNPs in the distal RU and the flanking pLAM region. They were consistent with the haplotype originally denoted as 4qC166H of unknown significance (Supplementary Fig. 7). As the definition of subhaplotypes and hybrid repeat arrays was based on individual samples and different regions, their overlap and variability are currently unknown and require harmonization.

We determined a distal methylation of the 4qA166 repeat array of 60%, which is above the FSHD-positive interval in this study (18–48%). The methylation profile showed a relatively high methylation in the proximal RU (29%) and a methylation gradient’s slope comparable to that in FSHD1 patients (4.0%/RU). A contraction of a 4qA166 repeat array, in combination with high distal methylation, strongly supported the exclusion of FSHD in this patient, in line with the atypical clinical phenotype and fast disease progression. Despite comprehensive analysis including exome and genome sequencing, analysis of mitochondrial genes and additional histopathological examination, the final diagnosis remained elusive.

#### Carrier status detection in a multi-ethnic family with South Asian population background

The complexity of interpreting multiple genetic parameters is exemplified by family G, which has a South Asian population background (referred to as ‘FSHD_South Asian population_’). The father (G1) presented in his sixties with a clear FSHD phenotype, as the first affected individual in his family, and would have been diagnosed as affected by FSHD1+2 based on distal and global hypomethylation in FSHD-MPA and the detection of a permissive 7 RU repeat array by OGM. However, the average size of all 4q35 and 10q26 repeat arrays was rather short compared to FSHD2*_SMCHD1_* patients (12 RU versus 20 RU on average), making this case suspicious of a potential artificial lowering of global methylation (Supplementary Fig. 2). A variant of unknown significance (VUS) was detected in *DNMT3B* by NGS, with a predicted low impact on *DNMT3B* protein function. Distal methylation determined by LRS was 40%, falling within the FSHD-positive interval. The methylation gradient’s slope was 3.3%/RU, within the range of FSHD1 patients in Cohort 1. Final differentiation between FSHD1 and 2 was not possible, also due to the lack of reference values for repeat length and methylation in this population. Exome sequencing to exclude differential diagnoses was negative.

Predictive testing of his daughter (patient G3) was complicated by (i) unknown penetrance of 7 RU repeat contraction in this population; (ii) unconfirmed validity of the detected global hypomethylation in her father and unknown pathogenicity of his *DNMT3B* variant; and (iii) insufficient validation of methylation assays for predictive testing. High correlation coefficients for distal methylation and clinical severity are observed only after age-adjustment of the latter. This suggests lifetime stability of DNA methylation as a prerequisite for predictive testing. For the most precise assessment of the disease risk of patient G3, all parameters were determined collectively. Based on global methylation in the intermediate range, distal methylation in the pathogenic range and the presence of a permissive repeat contraction of 9 RU, the daughter was diagnosed as being at risk of developing FSHD during her lifetime. Consistently, careful clinical examination after genetic testing revealed facial weakness as an initial sign of FSHD. Interestingly, the 9 RU repeat array was inherited from the mother (patient G2), who showed normal global and distal methylation and no obvious clinical signs of FSHD despite her higher age.

#### FSHD due to an 11 RU contraction in absence of global hypomethylation (FSHD_11 RU_)

Patient D1, affected by FSHD, had been misdiagnosed with an inflammatory myopathy for more than five decades. Following the revised diagnostic guidelines, she would also have been classified as negative for FSHD.^5^ The patient presented with discrete facial weakness, a foot drop palsy and mild proximal weakness, more pronounced in the lower than in the upper limb (Supplementary Table 3). Diagnostic criteria of FSHD1 (presence of a permissive repeat contraction ≤10 RU) were not fulfilled as the patient carried a permissive repeat array of 11 RU. Similarly, criteria of FSHD2 were not fulfilled based on the absence of likely pathogenic and pathogenic variants (LP/P) in known epigenetic suppressor genes (*SMCHD1*, *DNMT3B* and *LRIF1*) and global methylation above the pathogenic range.^5^

FSHD-MPA revealed reduced distal methylation within the pathogenic and global methylation within the intermediate range. Therefore, FSHD-MPA allowed the diagnosis of FSHD with inconclusive differentiation between FSHD1 and FSHD2.

Exome sequencing and repeat analysis for myotonic dystrophy types 1 and 2 did not identify an alternative origin of symptoms. Probatory immunosuppressive treatment did not improve the condition.

#### FSHD2 without a causative variant in known epigenetic suppressor genes (FSHD2_no variant_)

As no variants (SNVs and CNVs) in known epigenetic suppressor genes were identified by NGS, six patients (E1–E6) could only be identified as affected by FSHD2 based on FSHD-MPA results (Supplementary Tables 3 and 4). Four patients (E1, E2, E5 and E6) had typical FSHD phenotypes (ACSS: range 48–157), while two patients (E3 and E4) showed additional symptoms uncommon for FSHD. Patient E3 presented with a right-sided scapula alata since birth. In line with the diagnosis of a perinatal Erb’s palsy, EMG of the right shoulder girdle revealed neurogenic changes, whereas the left side was normal. However, progressive bilateral weakness of the shoulder girdle developed during adolescence, which was inconsistent with an isolated perinatal trauma and raised the suspicion of FSHD. Patient E4 displayed FSHD features in addition to early hypotonia, mild motor delay and exercise intolerance. All six patients (E1–E6) had negative family histories for FSHD and other neuromuscular disorders. All patients fulfilled the diagnostic criteria of FSHD2, including global hypomethylation and the presence of at least one permissive repeat array ≥8 RU. We compared the average repeat size of the four 4q35 and 10q26 repeat arrays in FSHD2_no variant_ and FSHD2*_SMCHD1_* patients, as repeat length can bias the global methylation level. Generally, FSHD2_no variant_ patients showed shorter mean average lengths (16 ± 2.6 RU, range 13–21 RU) than FSHD2*_SMCHD1_* patients (20 ± 5.6 RU, range 14–32 RU, *P* = 0.10). However, differences were not significant and both groups overlapped. FSHD disease status is confirmed by distal hypomethylation in the pathogenic (four patients) or intermediate range (two patients, both homozygous for the permissive haplotype 4qA).

We characterized both 4q35 repeat arrays in FSHD2_no variant_ patient E2 using ONT LRS. The shorter 16 RU 4qA repeat array had a methylation gradient’s slope of 1.2%/RU. Distal methylation was 41% and, as such, within the FSHD-positive interval determined in Cohort 1. Interestingly, the distal methylation of the second repeat array (19 RU on 4qAL) had a similar slope (1.4%/RU) but a relatively high distal methylation of 60%, despite the small length difference of 3 RU.

To investigate the origin of global hypomethylation, we first performed MLPA analysis of *SMCHD1* to detect CNVs that might have been missed by NGS, which was negative in all patients (E1–E6). SVs and CNVs in genes including *SMCHD1*, *DNMT3B* and *LRIF1* were further assessed via *de novo* assembly of OGM data in five patients (E1–E5) with suitable material available. No such variants were identified in any of these patients. Additionally, screening of previously identified FSHD2 candidate genes (*CTCF*, *DNMT1*, *DNMT3A*, *EZH2* and *SUV39H1*) from NGS data (patients E1–E6) revealed no potentially pathogenic variants.^22^ In patient E4, additional short-read whole-genome sequencing did not reveal a cause of global hypomethylation, but two compound heterozygous LP/P variants in *DOK7*. This establishes a dual diagnosis with congenital myasthenic syndrome (CMS), explaining atypical symptoms overlapping with FSHD.

### Upper threshold of repeat length in FSHD2*_SMCHD1_* patients

Beyond the complexity of rare genotypes, the genetic basis of FSHD2 remains incompletely understood. FSHD2 follows a digenic mode of inheritance, requiring an LP/P variant in *SMCHD1*, *DNMT3B* or *LRIF1* or global hypomethylation of other origin, in combination with a permissive repeat array with a size in the lower FSHD1-negative range. Current diagnostic guidelines assume 20 RU as the upper length threshold in most FSHD2 cases.^5^ To examine whether this threshold also applies to FSHD2*_SMCHD1_* in this study (patients B2–10), we determined the repeat lengths using OGM (Supplementary Table 4). The shorter 4qA repeat array ranged from 11 to 27 RU (average 19 ± 6 RU), with five of nine patients exceeding 20 RU, in line with previous reports.^20,32,33^ The high number of repeat arrays larger than 20 RU was partly influenced by the familial case of patients B5 and B6 (mother and son). Notably, these patients illustrate how a large second repeat array can bias global methylation. Patient B5 (second repeat array: 16 RU on 4qB) had a pathogenic global methylation, whereas patient B6 (second repeat array: 37 RU on 4qB) showed global methylation in the intermediate range. Both patients had a dual diagnosis of FSHD2 and *EYA4*-associated progressive bilateral sensorineural hearing loss since childhood. Of the five patients with more than 20 RU on their shorter 4qA-repeat array, four had a common FSHD phenotype indistinguishable from that of other FSHD2*_SMCHD1_* patients. The fifth patient (patient B6) had a relatively mild phenotype without facial weakness, as previously described.^34^ Average ACSS for all other FSHD2*_SMCHD1_* patients was similar for those with less or more than 20 RU on their shorter repeat array (140 ± 49 versus 141 ± 83, *P* > 0.05).

### Correlation of allele-specific distal methylation and age-corrected clinical severity

We further analysed the correlation between allele-specific distal methylation determined across the complete distal RU by ONT LRS and age-corrected clinical severity for patients of Cohort 1 and patients E2 and G1 of Cohort 2. Patients A7 and B1 were excluded due to incomplete clinical data; patient A8 was excluded due to somatic mosaicism of the contracted repeat array. For these 13 patients, a Pearson’s correlation coefficient of −0.81 (95% CI: −0.48 to −0.94) was determined (Fig. 2E). The threshold for pathogenic distal methylation was estimated at ≤48% based on the x-intercept of the function (ACSS = 0).

## Discussion

Diagnosing FSHD remains challenging due to its biological complexity and technical limitations. However, correct identification of patients affected by FSHD is not only important in the view of upcoming therapeutic options, but also to avoid injurious therapies based on an incorrect differential diagnosis. Interestingly, in at least four FSHD patients in this study, inflammatory myositis was considered as the disease origin after an initial negative genetic result for FSHD. In all patients, immunosuppressive therapy was initiated, likely causing liver fibrosis in one patient and facilitating active tuberculosis and non-Hodgkin lymphoma in another. In addition, the diagnosis cannot be based solely on the presence of a classic clinical phenotype, as dual diagnoses, as observed in our study (patients E3, E4 and B5, B6), are common and associated with atypical symptoms. Furthermore, atypical FSHD phenotypes, as part of the disease spectrum, require reliable molecular markers to confirm the diagnosis.

By enabling allele-specific analysis of the methylation profile of individual repeat arrays, LRS overcomes a key limitation of current methylation assays, in line with previous reports. Our study further supports distal methylation as a superior diagnostic parameter for determining overall disease status, as indicated by the following findings: (i) a strong correlation of allele-specific distal methylation with age-corrected disease severity was observed. (ii) Methylation levels of disease-causing and non-pathogenic repeat arrays in FSHD1 patients were completely separated. This allowed independent confirmation of FSHD1 in patients A1, A5 and A6, which had an inconclusive result in FSHD-MPA due to homozygosity for the permissive haplotype. (iii) Disease status was correctly determined based on distal methylation in all patients of Cohort 2, in contrast to a diagnostic only based on repeat length and variants in epigenetic suppressor genes. We propose the slope of the methylation gradient (Figs 1A and 2C) as an alternative parameter to differentiate FSHD1 from FSHD2, potentially replacing global methylation in BSS-based assays. In this study, FSHD1 patients (slopes of 2.5–8.1%/RU) were separated from the two FSHD2 patients in Cohort 1 (slopes of 0.3 and 1.2%/RU) based on the slope, consistent with a previous study reporting slopes of 1.9–3.9%/RU in FSHD1 and 0.7–1.6%/RU in FSHD2.^14^ As the slope is largely independent of the average repeat length of the 4q35 and 10q26 repeat arrays, as opposed to global methylation (illustrated in Supplementary Fig. 2), it may serve as a quantitative marker for the strength of epigenetic modifiers. Given the small sample size of Cohort 1, confirmation of our findings in larger and genetically diverse cohorts is required to validate the slope of the methylation gradient as a diagnostic parameter.

The methods currently used to diagnose FSHD each have distinct advantages and limitations (Supplementary Table 10). As illustrated by patient F1, OGM does not detect intra-array SVs, as labelling occurs only proximal and distal to the 4q35 and 10q26 repeat arrays. This leads to misclassification of 4qA166 repeat arrays or SVs such as *in cis* duplications and 4q-10q rearrangements. Patient F1 showed rapid disease progression and severe respiratory insufficiency, which are atypical for FSHD. However, initial symptoms were compatible with the disease. Together with the detection of a contracted repeat array on an apparently permissive and pathogenic repeat array by OGM, this could have led to a false FSHD diagnosis. Based on high distal methylation (60%) exceeding the FSHD-positive interval, FSHD was correctly excluded by ONT LRS. Additionally, the patient carried the 4qA166 haplotype *in cis* to the repeat contraction, which is currently not associated with FSHD despite carrying a PAS. Non-pathogenicity of 4qA166 repeat arrays was derived in previous studies from two older, unrelated and unaffected carriers of 4qA166 repeat arrays with 5 and 8 RU.^35^ Patient F1 in our study showed a methylation gradient slope similar to FSHD patients, but an unusually high proximal methylation. The first six RUs showed average methylation within the FSHD1-positive interval, implying that a similar, but shorter, repeat array might show distal methylation in the pathogenic range. As such, general non-pathogenicity of 4qA166 repeat arrays should be assumed with caution. It may, in principle, be possible that lower cut-offs exist for the pathogenicity of 4qA166 repeat arrays.

The challenges of assessing the pathogenicity of permissive and contracted repeat arrays based solely on repeat length, particularly in underrepresented population backgrounds, are exemplified by family G. In Asian populations, different thresholds for FSHD1 have been reported, e.g. 1–6 RU in Korea and Japan or 1–9 RU in China.^36-38^ The 7 RU repeat array of the index patient (G1) was classified as pathogenic only through the additional finding of distal hypomethylation. Likewise, the disease risk of his daughter (G3) could only be established by detecting distal hypomethylation of the 9 RU repeat array inherited from the mother (G2). In the mother, the 9 RU repeat array showed a distal methylation within the normal range, in line with the absence of clinical signs of FSHD despite her higher age. Results from this family indicate that methylation might represent a more general parameter of disease status than repeat length and may be less dependent on population background. However, the applicability of identical methylation thresholds across different populations requires validation in larger and more diverse cohorts. The findings in family G further indicate the presence of epigenetic modifiers causing pathogenicity of the maternally inherited 9 RU repeat array. This is also reflected by the pronounced distal hypomethylation in patient G3, in contrast to her mother. Although the daughter is heterozygous for the permissive haplotype while her mother is homozygous, it is unlikely that the second permissive repeat array of the mother masks pathogenic hypomethylation of her 9 RU repeat array. If that were the case, we would expect a result in the intermediate rather than in the normal range. As the quantitative relationship between global methylation and repeat size is currently unknown, the ability to conclude the presence of epigenetic modifiers based on reduced global methylation in the index patient and his daughter compared to the mother is limited, also given the larger average 4q35 and 10q26 repeat length (16 RU) in the mother compared to the index patient and his daughter (12 RU). Whether the variant detected in *DNMT3B* serves as the respective epigenetic modifier remains unknown, with bioinformatic predictions rather indicating non-pathogenicity. The slope of the methylation gradient of 3.3% in the index patient does not support the presence of strong but rather moderate epigenetic modifiers, although reference values for different populations remain to be established.

The pathogenicity of permissive repeat arrays of 11 RU in the absence of global hypomethylation remains controversial. While in some cases they were regarded as borderline with reduced penetrance, current diagnostic guidelines define the FSHD1 cut-off at 10 RU for the European and North American populations.^5,39^ In this study, we report one patient with an FSHD phenotype (patient D1) carrying a permissive repeat array of 11 RU. Based on global hypomethylation within the intermediate range, we hypothesize the presence of additional weak to moderate epigenetic modifiers contributing to pathogenicity.

Cases of FSHD2 marked by global hypomethylation but without detectable variants in known epigenetic suppressor genes are well described and account for up to 15% of FSHD2 patients.^2,4,10,40^ Similarly, in our study, the cause of global hypomethylation remained elusive in six FSHD2_no variant_ patients (patients E1–E6). These cases highlight the need to routinely assess global methylation, as recommended in the revised diagnostic guidelines.^5^ Restricting FSHD2 testing to sequencing of known epigenetic suppressor genes, as still practised in many diagnostic laboratories, may miss such cases. Given the substantial number of FSHD2 patients with an unknown cause of hypomethylation, deriving the pathogenicity of VUS in *SMCHD1* based solely on co-occurrence with global hypomethylation should be performed with caution. This is particularly critical in prenatal and preimplantation diagnostics, where accurate variant classification is essential, since methylation patterns likely change during early embryonic development and are not valid as diagnostic parameters.

In principle, missed SVs, deep intronic or regulatory variants in *SMCHD1*, *DNMT3B* and *LRIF1*, or other candidate genes could explain global hypomethylation in FSHD2_no variant_ patients. In these cases, long-read whole-genome and RNA sequencing of blood and muscle could further be applied to search for causative variants. However, given the broad variety of methods applied, this appears unlikely in all FSHD2_no variant_ cases. Alternatively, currently unknown epigenetic suppressor genes could exist. Interestingly, patients with FSHD2_no variant_ had, on average, shorter 4q35 and 10q26 repeat arrays than patients with FSHD2*_SMCHD1_*. However, it is unlikely that the reduced global methylation observed in FSHD2_no variant_ patients is only a technical artefact of the shorter repeat arrays, as the average repeat lengths of both groups largely overlap. Rather, we hypothesize that weaker epigenetic modifiers in FSHD2_no variant_ patients are present that can only be detected in the absence of large 4q and 10q repeat arrays, which would artificially increase global methylation. That epigenetic modifiers in FSHD2_no variant_ patients are weaker than in FSHD2*_SMCHD1_* patients is supported by (i) a larger slope of the methylation gradient in FSHD2_no variant_ patient E2 (1.2%) compared to FSHD2*_SMCHD1_* patient B1 (0.3%) and (ii) two FSHD2_no variant_ patients having a distal methylation in the intermediate range, which is atypically high for FSHD2, even in patients being homozygous for the permissive haplotype. However, additional FSHD2*_SMCHD1_* and FSHD2_no variant_ patients need to be analysed by LRS to confirm these differences. It additionally needs to be studied whether FSHD2_no variant_ patients might on average show milder phenotypes.

Both FSHD2_no variant_ and FSHD_11 RU_ patients are equally a source of ‘clinical FSHD, genetic cause unknown’ diagnoses as defined in the updated diagnostic guidelines.^5^ Especially when large 4q35 and 10q26 repeat arrays are present, moderate to weak epigenetic modifiers of global hypomethylation might be masked. In such cases, global and distal methylation might be in the intermediate range and diagnosis of FSHD remains elusive. This is observed in a small number of patients with distinct FSHD phenotype in our institution (data not shown). We hypothesize that SNPs not fulfilling the classification of an LP/P variant within genes responsible for methylating the 4q35 repeat array could act as weak and moderate epigenetic modifiers by potentially reducing the efficiency of the associated gene. In FSHD2_no variant_ patients, the accumulation of multiple such variants may exert a combined effect on the overall methylation similar to an LP/P variant in known epigenetic suppressor genes. Such an oligogenic mode of inheritance would be supported by the negative family history of all FSHD2_no variant_ patients. Weak to moderate epigenetic modifiers could not only explain the pathogenicity of some repeat arrays with a size of 11 RU, but also why repeat contractions of 8–10 RU are penetrant in only a subset of individuals of the European and North American populations.^41,42^ SNPs in epigenetic suppressor genes with opposite effects might also explain lower pathogenic thresholds for FSHD1 in other populations, as certain variants in *SMCHD1* are associated with higher methylation levels of the 4q35 repeat array.^43^ In addition, variants in epigenetic regulators influencing chromatin structure are candidates that impact disease status in ways not reflected by DNA methylation.

While the relationship between DNA methylation and empirically identified genetic parameters (4q35 repeat contractions and LP/P variants in epigenetic suppressor genes) has remained incompletely understood, it can now be elucidated by an asymmetric methylation gradient at 4q35 and 10q26 repeat arrays (Figs 1A and  3).^13-15^ We propose that repeat length, LP/P variants in known epigenetic suppressor genes and unknown epigenetic modifiers collectively shape the methylation profile of a D4Z4 repeat array. A hypothesis-driven model in line with our observations is presented in Fig. 3. In FSHD1 patients A1–A12 in our study, the average methylation within the most proximal RU is 19% and increases by on average 4.8% per RU. As such, distal methylation would on average exceed the FSHD-positive interval of 18–48% after the 7th RU, which equals the known repeat length threshold for fully penetrant repeat arrays in FSHD1 in the studied population. Variability in the methylation gradient’s slope (range: 2.5–8.1%/RU; average: 4.8 ± 1.8%/RU) likely reflects the influence of weak and moderate epigenetic modifiers. Consequently, distal methylation thresholds are reached after a varying number of RUs, potentially explaining inter-individual and population-specific repeat length thresholds in FSHD1. Additionally, this could explain variations in disease severity among patients with similar repeat lengths.

**Figure 3:**
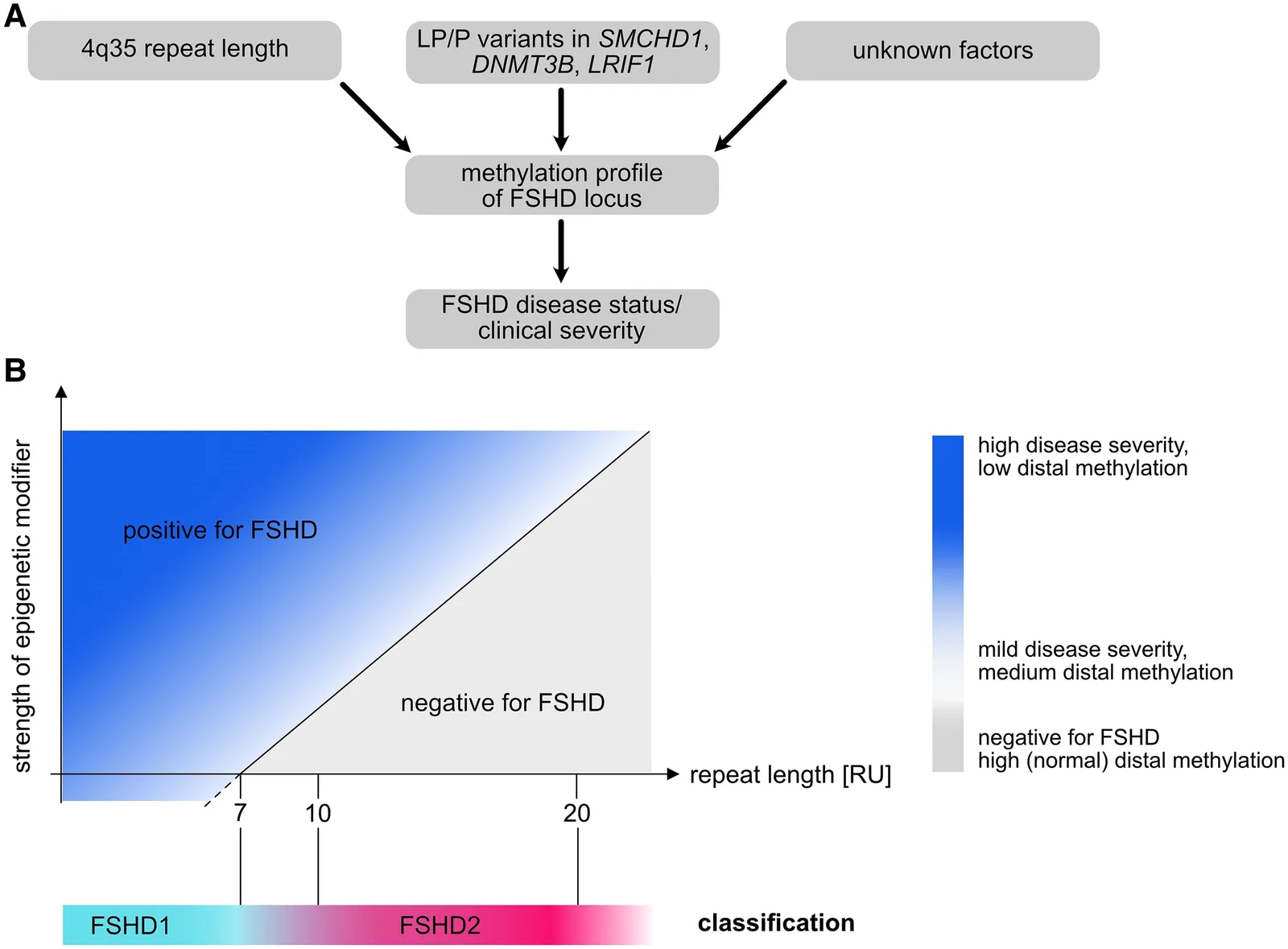
**Linkage of genetic parameters, epigenetics (methylation) and phenotype in FSHD.** (**A**) Hypothesis-driven model of repeat length, LP/P variants in epigenetic suppressor genes and unknown epigenetic modifiers impacting the methylation profile of the FSHD locus linked to disease status. (**B**) Hypothesized model of the proposed impact of repeat length (*x*-axis) and strength of epigenetic modifiers (*y*-axis) on distal methylation as a quantitative biomarker for FSHD disease status. In previous studies, low distal methylation levels were, on average, associated with a severe FSHD phenotype (shown in deep blue, upper left area), while medium distal methylation levels were, on average, associated with a mild FSHD phenotype (shown in white).^11,12^ High distal methylation levels were associated with the absence of FSHD (shown in grey, lower right area). In our model, short repeat arrays and strong epigenetic modifiers are proposed to cause low distal methylation levels (shown in deep blue). Following this hypothesis, the longer the repeat array and the weaker the epigenetic modifiers, the higher the distal methylation. At a certain point (marked by the black line within the diagram), distal methylation levels cross the border of the pathogenic and normal, non-pathogenic range. Strong epigenetic modifiers can be LP/P variants in *SMCHD1*, while the nature of weak modifiers is currently unknown and could hypothetically be resembled by non-LP/P variants in epigenetic suppressor genes. The dashed line indicates hypothetical inverse epigenetic modifiers that potentially increase distal methylation and might explain lower thresholds of pathogenicity in other populations. The bottom scale indicates FSHD as a molecular continuum with FSHD1 and FSHD2 as the two extremes. FSHD1 is primarily caused by short repeat arrays (1–7 RU in the European population), while FSHD2 is primarily caused by epigenetic modifiers such as LP/P variants in *SMCHD1*, *DNMT3B* and *LRIF1* in FSHD2. Both are likely relevant in the case of individuals affected by FSHD carrying about 8–10 RU. 20 RU is assumed as the upper threshold for pathogenicity in most FSHD2 patients; however, larger permissive repeat arrays can hypothetically lead to FSHD manifestation in case of strong epigenetic modifiers.

The presence of pathogenic variants in known epigenetic suppressor genes as strong epigenetic modifiers likely causes a flatter gradient’s slope in FSHD2. Consequently, methylation levels in the non-pathogenic range are likely reached after a higher number of RU (Figs 1A and 3B). This suggests the existence of an upper threshold for repeat length in FSHD2, which may differ depending on the origin of global hypomethylation with respect to the specific variant and the epigenetic suppressor gene. A recent study confirmed that *SMCHD1* variants differ in their impact on global methylation levels without identifying differences depending on the type of variant.^20^ We hypothesize that variants causing strong global hypomethylation, potentially reflected in low slopes of the methylation gradient, lead to FSHD manifestation even with longer permissive repeat arrays (e.g. 27 RU in this study), while milder variants and modifiers associated with steeper gradients would require shorter permissive repeat arrays to cause FSHD, possibly the case in FSHD2_no variant_ patients. This proposed model is in line with the concept of FSHD as a molecular continuum (Fig. 3B), where repeat length predominates in FSHD1 (1–7 RU in the European and North American populations) and epigenetic modifiers are primary drivers in FSHD2. Both factors likely interact in the incomplete penetrance range (8–10 RU in the European population), in FSHD patients with 11 RU in the absence of global hypomethylation and potentially some FSHD2_no variant_ patients.

While our study presents LRS as a superior approach to study the FSHD locus, the presented workflow cannot yet be used as a diagnostic test for several reasons: (i) the number of samples analysed with LRS in our study is limited. (ii) Target enrichment via CRISPR/Cas9 is largely incompatible with current ONT R10 flow cells. This, together with the limited availability of sufficient amounts of high molecular weight DNA, is the reason why only three patients of Cohort 2 underwent LRS. Future studies should focus on alternative strategies for target enrichment, e.g. adaptive sampling as presented in other studies.^44-46^ (iii) The method and the methylation gradient’s slope as a diagnostic parameter require further validation regarding reproducibility, stability, accuracy and required minimum sequencing depth. (iv) Accuracy and limits of detection need to be determined for repeat contractions present as somatic mosaicism and complex structural variants. (v) The determined cut-off values require refinement in larger cohorts with diverse population backgrounds and controls not affected by a neuromuscular disease.

We summarized the advantages and disadvantages of current standard diagnostic methods in Supplementary Table 10. Based on this analysis and the results of our study, we recommend performing FSHD-MPA or similar assays according to our previously described workflow, with confirmatory repeat length analysis for FSHD1 and/or sequencing of epigenetic suppressor genes for FSHD2.^12^ In case of a negative result (global and distal methylation in the normal range), despite a clear FSHD phenotype, Southern blot with pulsed-field gel electrophoresis and different probes should be performed to test for somatic mosaicism and indicators of rearrangements and other SVs.^12^ In case of negative results following a traditional diagnostic workflow with repeat length analysis and sequencing of epigenetic suppressor genes as first-tier analyses, global methylation should routinely be analysed when a permissive haplotype has been detected. However, as a long-term goal, implementation of long-read sequencing as a stand-alone, first-tier approach for FSHD diagnostics should be pursued when technically and economically available.

## Supplementary Material

fcag370_Supplementary_Data

## Data Availability

The data underlying from this article are available from the corresponding author on reasonable request. The code used for the downsampling analysis and statistical analyses is provided as Supplementary Code S1. The code for filtering reads is based on the published method of Butterfield *et al*.^14^
