# Supplementary material for: Decoding common and complex genotypes of facioscapulohumeral muscular dystrophy using methylation profiling and long-read sequencing
Source: Brain Commun. 2026 Oct 10;8(5):fcag370. doi: 10.1093/braincomms/fcag370 (PMC13685627; doi:10.1093/braincomms/fcag370)
Supplement: Supplementary file 1 [file fcag370_supplementary_data.zip › Supplementary Material_R3.pdf]

Supplementary Material

Scharf and Lucas et al.

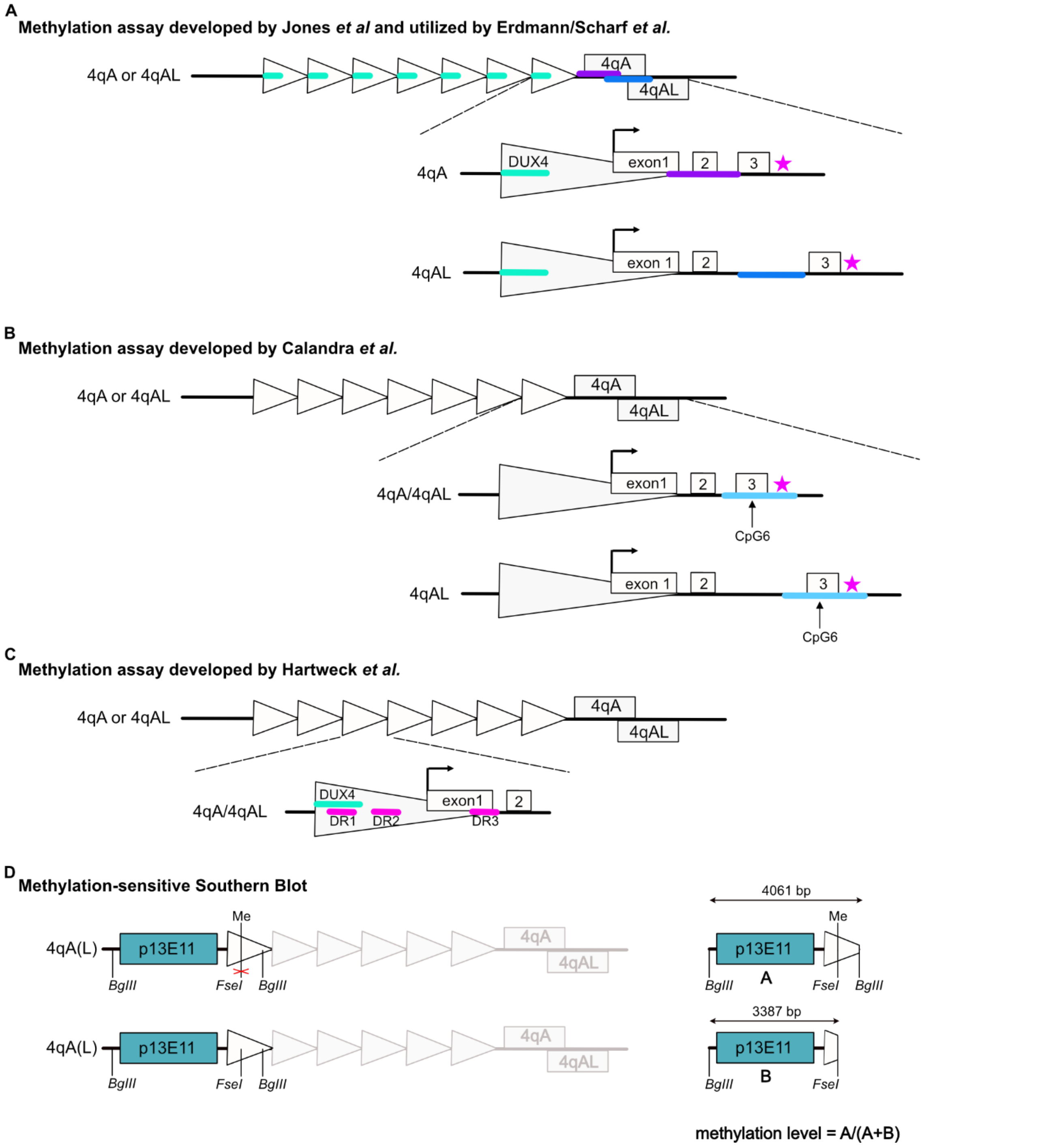

Supplementary Figure 1. Overview of the different methylation assays used in FSHD diagnostics. (A) Methylation profile analysis (FSHD-MPA) developed by Jones *et al.*[1] and adopted by Erdmann and Scharf *et al.*[2] Distal methylation levels of 4qA (pink) and 4qAL alleles (blue) are used to determine overall FSHD disease status. The pink star highlights the SNP that encodes for a polyadenylation signal defining the permissive haplotype. In homozygous patients, distal methylation represents the average of both alleles. Average methylation of all D4Z4 repeat units of chromosome 4q35 and chromosome 10q26 represents 'global methylation' (turquoise) and is used to differentiate FSHD1 from FSHD2. In FSHD-MPA repeat arrays of chromosome 4q35 are preferentially amplified. (B) Methylation assay of Calandra *et al.*[3] also measures methylation within the most distal repeat unit. The region assayed is equally present in permissive 4qA and 4qAL repeat arrays. Allele-specific information is also lost in homozygous individuals, and the assay does not distinguish between FSHD1 and FSHD2. (C) The methylation assay developed by Hartweck *et al.*[4] determines global methylation levels across D4Z4 repeat arrays on both chromosome 4q35 and 10q26 and targets the DR1 region, which overlaps with the region analyzed in the DUX4 assay in FSHD-MPA. DR2 and DR3 were investigated but are not used in current diagnostics. They show higher methylation due to periodic changes in the methylation patterns within a single repeat unit as now revealed by LRS. In contrast, DR1 corresponds to a hypomethylated region at the start of one repeat unit. (D) Methylation-sensitive restriction digest with *FseI* prior to Southern blotting assesses average methylation within the most proximal repeat unit of chromosome 4q35 and 10q26.[5] Additional digestion with *BlnI* eliminates fragments from chromosome 10q26. While proximal methylation distinguished FSHD2 from FSHD1 and controls in previous studies, it is less effective at distinguishing FSHD1 from controls.

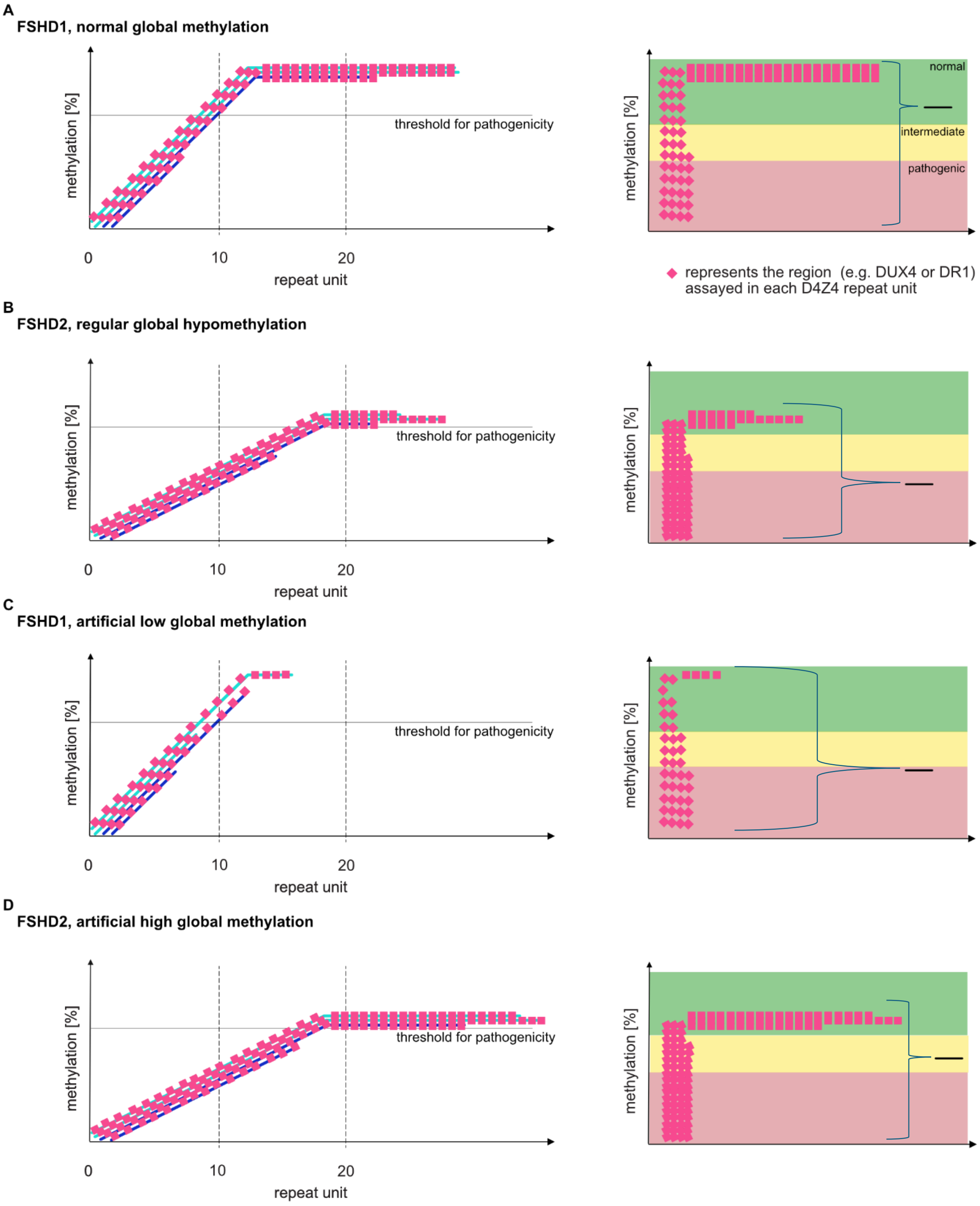

Supplementary Figure 2. Potential biases in determining global methylation in standard methylation assays in light of the asymmetric methylation gradient. Pink dots represent the regions assayed in each repeat unit of 4q35 and 10q26 repeat arrays in order to determine the average methylation level (referred to as 'global methylation' and represented as black bar, right graphs). Dark blue lines (bottom lines, left graphs) represent repeat arrays on chromosome 4q35 and light blue lines represent repeat arrays on chromosome 10q26 (top lines). The representation of the methylation pattern takes into account the presence of an asymmetric methylation gradient, that increases from the proximal to the distal end and is flatter in FSHD2 than in FSHD1 patients. (A) Normal result of global methylation (high average methylation) in FSHD1 patients and non-affected individuals (not shown). (B) Global methylation within the pathogenic range (low average methylation) in FSHD2 patients. (C) Artificially reduced global methylation due to additional short repeat arrays on 4q35 and 10q26 potentially leading to global methylation in the pathogenic or intermediate range in FSHD1 patients. This is especially relevant for the FSHD-MPA method in case of additional short 4q35 repeat arrays, as those are preferentially amplified. (D) Artificial dilution of global hypomethylation of the pathogenic allele in FSHD2 patients by large repeat arrays on the non-disease causing alleles. As a consequence, global methylation is within the intermediate range and inconclusive.

Supplementary Table 1. Overview of patients analyzed in this study including methods used to study their genotype, assignment to the cohorts, their sex and final diagnosis.

	A1	A2	A3	A4	A5	A6	A7	A8	A9	A10	A11	A12	B1	C1	D1	E1	E2	E3	E4	E5	E6	F1	G1	G2	G3	B2	B3	B4	B5	B6	B7	B8	B9	B10
BSS-methylation assay (FSHD-MPA)	■	■	■		■	■	■	■	■	■	■	■	■	■	■	■	■	■	■	■	■	■	■	■	■	■	■	■	■	■	■	■	■	■
Optical Genome Mapping (repeat length analysis)	■	■			■	■		■			■			■	■	■	■	■	■	■		■	■	■	■	■	■	■	■	■	■	■	■	■
Southern blot (p13E11 probe, LGE)				■			■	■	■	■	■		■		■						■	■												
Additional haplotype analysis (KASP & Tsumagari)	■	■	■	■	■	■	■	■				■	■	■	■	■	■	■	■	■	■	■	■	■	■	■	■	■	■	■	■	■	■	■
NGS (*SMCHD1*, *DNMT3B*, *LRIF1*)													■	■	■	■	■	■	■	■	■	■	■	■	■	■	■	■	■	■	■	■	■	■
MLPA (*SMCHD1*)														■		■	■	■	■	■	■							■						
Optical Genome Mapping (*SMCHD1*, *DNMT3B*, *LRIF1*)														■		■	■	■	■	■														
NGS of FSHD2 candidate genes*														■		■	■	■	■	■	■													
NGS (whole exome sequencing)													■	■	■	■	■	■	■	■	■	■	■	■	■	■	■	■	■	■	■	■	■	■
Repeat analysis *CNBP/DMPK*															■							■												
NGS (whole genome sequencing)																			■															
FSHD analysis by ONT LRS	■	■	■	■	■	■	■	■	■	■	■	■	■	■			■					■	■											
Cohort	Cohort 1	Cohort 1	Cohort 1	Cohort 1	Cohort 1	Cohort 1	Cohort 1	Cohort 1	Cohort 1	Cohort 1	Cohort 1	Cohort 1	Cohort 1	Cohort 1	Cohort 2	Cohort 2	Cohort 2	Cohort 2	Cohort 2	Cohort 2	Cohort 2	Cohort 2	Cohort 2	Cohort 2	Cohort 2	Cohort 2	Cohort 2	Cohort 2	Cohort 2	Cohort 2	Cohort 2	Cohort 2	Cohort 2	Cohort 2
Sex	F	M	M	M	M	F	F	M	F	M	F	F	M	F	F	F	M	M	M	M	F	F	M	F	F	M	M	F	F	M	F	M	M	M
Diagnosis	FSHD1	FSHD1	FSHD1	FSHD1	FSHD1	FSHD1	FSHD1	FSHD1	FSHD1	FSHD1	FSHD1	FSHD1	FSHD2$_{SMCHD1}$	FSHD1+2$_{\text{no variant}}$	FSHD$_{11\ RU}$	FSHD2$_{\text{no variant}}$	FSHD2$_{\text{no variant}}$	FSHD2$_{\text{no variant}}$	FSHD2$_{\text{no variant}}$	FSHD2$_{\text{no variant}}$	FSHD2$_{\text{no variant}}$	negative for FSHD, unknown muscle disease	FSHD$_{\text{South Asian population}}$	negative for FSHD, no signs of muscle dystrophy	FSHD$_{\text{South Asian population}}$	FSHD2$_{SMCHD1}$	FSHD2$_{SMCHD1}$	FSHD2$_{SMCHD1}$	FSHD2$_{SMCHD1}$	FSHD2$_{SMCHD1}$	FSHD2$_{SMCHD1}$	FSHD2$_{SMCHD1}$	FSHD2$_{SMCHD1}$	FSHD2$_{SMCHD1}$

Abbreviations: BSS: bisulfite sequencing; LGE: linear gel electrophoresis; KASP: Kompetitive allele specific PCR; NGS: next-generation sequencing; MLPA: multiplex ligation-dependent probe amplification; ONT: Oxford Nanopore Technology; LRS: long-read sequencing

* FSHD2 candidate genes *CTCF*, *DNMT1*, *DNMT3A*, *EZH2*, *SUV39H1* as proposed by Strafella et al.[6]

- Cohort 1
- Cohort 2
- FSHD1
- FSHD2$_{SMCHD1}$
- FSHD1+2$_{\text{no variant}}$
- FSHD$_{11\ RU}$
- FSHD2$_{\text{no variant}}$
- negative for FSHD, unknown muscle disease
- FSHD$_{\text{South Asian population}}$
- negative for FSHD, no signs of muscle dystrophy
- ■ test was performed

Supplementary Table 2. Characterization of patients with common FSHD genotypes (Cohort 1) and three patients of Cohort 2 by CRISPR/Cas9-targeted Oxford Nanopore long-read sequencing. For each patient, ONT LRS provided data on repeat size and haplotype of the shorter allele, average methylation levels within the most proximal and most distal repeat unit, and the slope of the methylation gradient for the contracted allele. Additional data include results from haplotype analysis using the protocol of Tsumagari et al.[7] and a KASP assay detecting a single nucleotide variant within the most distal repeat unit of permissive alleles as previously described,[2] FSHD-MPA, Optical Genome Mapping and/or Southern blot analysis, and age-corrected clinical severity score (ACSS). '+' with regard to the KASP assay indicates presence of the permissive haplotype. For FSHD-MPA '+' indicates methylation level within the pathogenic range, '(+)' methylation level within the intermediate range and '–' methylation level within the normal range.

Patient (sex)	Haplotype analysis		FSHD-MPA						Repeat length determined by OGM and/or Southern blot[b]	Repeat length and haplotype determined by ONT LRS	ONT LRS				ACSS
	Tsum-agari assay[a]	KASP assay	DUX4 assay methylation [%]		4qA assay methylation [%]		4qAL assay methylation [%]				proximal methylation contracted/ shorter allele [%]	distal methylation contracted/ shorter allele [%]	methylation gradient's slope of the contracted/ shorter allele [%/RU]	distal methylation uncontracted/ larger allele [%]	
Cohort 1															
FSHD1															
A1 (female)	4qA161/ 4qA161	+	47.7	-	43.7	(+)	/	/	5 RU 4qA/ 26 RU 4qA	5 RU 4qA/ >24 RU 4qA	24	45	4.7	78	77
A2 (male)	4qA161/ 4qA161	+	55.2	-	/	/	52.3	+	7 RU 4qA/ 53 RU 4qA	7 RU 4qAL/ >11 RU 4qAL	19	34	2.5	ND[d]	191
A3 (male)	4qA161	+	66.6	-	22.7	+	73.8	-	ND	6 RU 4qA (70% of reads)/>25 RU 4qB (16% of reads)/>19 RU 4qAL (14% of reads)[c]	21 (4qA)	40 (4qA)	3.7	80 (4qAL), 85 (4qB)	69
A4 (male)	4qA161/ 4qA161	+	ND	ND	ND	ND	ND	ND	5 RU (SB)	5 RU 4qA/ ND	19	38	4.5	ND[d]	27
A5 (male)	4qA161/ 4qA161	+	57.4	-	36.5	(+)	/	/	6 RU 4qA/ 46 RU 4qA	6 RU 4qA/ >14 RU 4qA	18	34	2.7	81	116
A6 (female)	4qA161/ 4qA161	+	53.3	-	39.5	(+)	/	/	7 RU 4qA/ 32 RU 4qA	7 RU 4qA/ >11 RU 4qA	17	44	4.2	ND[d]	105
A7 (female)	4qA161	+	48.6	-	22.0	+	/	/	5 RU (SB)	5 RU 4qA/ >14 RU 4qA	19	34	3.9	77	NA
A8 (male)	4qA161	+	55.7	-	52.4	-	/	/	2 RU 4qA (somatic mosaicism in ~54% of cells)/ 47 RU 4qA/ 24 RU 4qB	2 RU (4qA)/ >14 RU 4qA/ >10 4qB (somatic mosaicism)	22	30	8.1	79	14
A9 (female)	ND	ND	47.2	-	12.3	+	/	/	2 RU (SB)	2 RU 4qA >7 RU 4qA	17	22	4.6	ND[d]	217

A10 (male)	ND	ND	55.5	-	14.9	+	/	/	2 RU (SB)	2 RU 4qA/ >16 RU 4qA	17	25	7.6	87	318
A11 (female)	ND	ND	50.2	-	11.8	+	/	/	2 RU 4qA/ 17 RU 4qA; 2 RU (SB)	2 RU 4qA/ 17 RU 4qA	19	27	7.4	77	333
A12 (female)	4qA161/ 4qB163	+	50.5	-	12.5	+	/	/	ND	5 RU 4qA/ND	10	24	3.3	ND[d]	333
FSHD2 with causative variant in SMCHD1 (FSHD2$_{SMCHD1}$)															
B1[e] (male)	4qA161/ 4qA161	+	34.5	+	16.3	+	/	/	11 RU (Southern blot)	11 RU 4qA/ND[c]	21	30	0.3	ND[d]	NA
FSHD1+2 without confirmed variant in SMCHD1, DNMT3B and LRIF1 (FSHD1+2$_{no\ variant}$)															
C1 (female)	4qA161/ 4qB163	+	27.2	+	29.6	+	34.1	+	7 RU 4qA 31 RU 4qA	6 RU 4qAL/ 31 RU 4qA/	19 (4qAL)/ 33 (4qA)	24 (4qAL)	1.2 (4qA)	52 (4qA)	172
**Cohort 2**															
FSHD2 without causative variant in SMCHD1, LRIF1, DNMT3B (FSHD2$_{no\ variant}$)															
E2 (male)	4qA161/ 4qB163	+	32.7	+	27.0	+	53.1	(+)	16 RU 4qA/ 19 RU 4qA	16 RU 4qA/ 19 RU 4qAL	24 (4qA)	41 (4qA)	1.2 (4qA), 1.4 (4qAL)	60 (4qA)	44
Non-pathogenic complex 4qA166 repeat array															
F1 (female)	4qA161	+	51.9	-	60.4	-	/	/	7 RU 4qA/ 43 RU 4qA	7 RU (4qA166)/ >11 RU (4qA)	29 (4qA166)	60 (4qA166)	4.0 (4qA166)	77 (4qA)	ND[f]
Complex genetic findings in light of a South Asian population background (FSHD$_{South\ Asian\ population}$)															
G1 (male)	4qA161/ 4qB163	+	36.9	+	17.7	+	/	/	7 RU 4qA/ 14 RU 4qB	7 RU 4qA/ 14 RU 4qB	20	40	3.3	ND[d]	87

Abbreviations: KASP: Kompetitive allele specific PCR; ONT: Oxford Nanopore Technology; LRS: long-read sequencing; OGM: Optical Genome Mapping; DD: differential diagnosis; NA: not available; ND: not determined; SB: Southern blot.

[a] Based on single nucleotide polymorphisms (SNPs) in the region proximal to the 4q35 D4Z4 repeat array following the protocol of Tsumagari *et al*. SNP combinations of 4qA166/4qB168/10qA166 are identical. Therefore, in some cases, it is not possible to distinguish whether patients are homozygous for 4qA161 and carry the 10qA166 haplotype or heterozygous for 4qA161 and carry the 4qB168 haplotype at the other allele. For these patients only one haplotype (4qA161) is given in the Table.
[b] OGM cannot distinguish the 4qA and 4qAL haplotype. In both cases, the result would be 4qA.
[c]Origin of three different 4qA35 repeat arrays remains unknown. In principle, the presence of one of the alleles at chromosome 10q26 due to a rearrangement or of two repeat arrays at one allele as *in cis* duplications or a somatic mosaicism is possible.
[d]Not determined due to insufficient coverage of the uncontracted allele.
[e]Detection of the likely pathogenic (ACMG), heterozygous variant NM_015295.3: c.38del p.(Ser13Leufs*96) in *SMCHD1*.
[f]Not determined as the patient was diagnosed negative for FSHD.

Supplementary Table 3. Clinical presentation of patients of Cohort 2. Age at examination, family history for FSHD, clinical signs of FSHD (e.g. myopathic face, scapula alata, polyhill sign, horizontal axillary fold, Beevor sign), involved muscles, creatine kinase levels, findings in EMG and other examinations, clinical diagnosis based on phenotype and age-corrected clinical severity score (ACSS).

Patient (sex)	Age [years]	Family history	Clinical signs of FSHD	Muscular weakness	Relevant additional symptoms/ diagnoses	CK level [U/L][a]	EMG	additional instrumental results	Clinical diagnosis	ACSS (CSS, range 0.5–5)
FSHD with 11 RU in absence of global hypomethylation (FSHD$_{11\ RU}$)										
D1 (female)	86	positive (daughter)	• asymmetric scapula alata	• discrete facial weakness<br>• mild proximal weakness of the upper and lower limb (lower limb > upper limb)<br>• foot drop palsy	-	96	NA	MRI: fatty degeneration of autochthonous and hamstring muscles<br>Histology: mixed chronic-neurogenic and necrotic myopathic changes	myopathy<br>DD atypical FSHD	70 (3)
FSHD2 without causative variant in SMCHD1, LRIF1, DNMT3B (FSHD2$_{no\ variant}$)										
E1 (female)	75	negative	• myopathic face<br>• scapula alata<br>• horizontal axillary fold<br>• hyperlordosis	• facial weakness<br>• proximal weakness of the upper and lower limb<br>• foot drop palsy	diabetic neuropathy	237 (age of 58)	myopathic changes, PSA, neurogenic changes	MRI of the lower limb: fatty degeneration of the vastus and hamstring muscle (age of 72)	FSHD	133 (5)
E2 (male)	45	negative	• horizontal axillary fold	• mild proximal weakness of the upper limb	-	293	normal	-	myopathy DD FSHD or *FHL1*-associated myopathy	ND[c]
E3 (male)	18	negative	• scapula alata<br>• Popeye sign	• asymmetric progressive proximal weakness of the upper limb	perinatal Erb's palsy (right side)	< 170	normal	-	FSHD possible	111 (1)
E4 (male)	9	negative	• myopathic face<br>• scapula alata	• proximal weakness of the upper and lower limb	hypotonia in the age of 3 years; mild motor delay	< 170	NA	Histology: unspecific myopathic pattern in muscle histology	myopathy	ND[c]
E5 (male)	42	negative	• myopathic face<br>• scapula alata<br>• hyperlordosis	• facial weakness<br>• mild proximal weakness of the upper limb<br>• axial weakness	-	< 170	myopathic changes	-	FSHD	48 (1)
E6 (female)	51	negative	• myopathic face<br>• scapula alata<br>• Beevor sign positive	• facial weakness<br>• proximal weakness of the	-	< 170	NA	-	FSHD	157 (4)

				upper and lower limb<br>• foot drop palsy						
Non-pathogenic complex 4qA166 repeat array									
F1 (female)	55	negative		• rapidly progressive proximal weakness of the upper and lower limb with axial weakness and severe respiratory insufficiency within three years	Sjörgen's syndrome	3–5x elevated	myopathic changes	MRI: fatty degeneration of the limb girdle and signs of myositis; Histiocytic inflammation and myopathic changes in muscle histology	myopathy DD FSHD	ND[b]
Complex genetic findings in light of an Asian population background (FSHD$_{South\ Asian\ population}$)									
G1 (male)	70	negative	• scapula alata<br>• Beevor sign positive	• mild facial weakness<br>• asymmetric proximal weakness of the of the upper and mild proximal weakness of the lower limb<br>• foot drop palsy	-	235	neurogenic and myopathic changes	-	FSHD	87 (3)
G2 (female)	60	negative	none apparent	• none apparent	-	NA	NA	-	no indication of FSHD	-
G3 (female)	30	negative	none apparent	• mild facial weakness	-	111	normal result	-	FSHD	33 (0.5)
FSHD2* with (likely) pathogenic *variant in SMCHD1 (FSHD2$_{SMCHD1}$)									
B2 (male)	23	negative	• scapula alata	• asymmetric proximal weakness of the upper limb and mild proximal weakness of the lower limb<br>• foot drop palsy	-	~340–500	neurogenic changes	Histology: myopathic changes	FSHD	261 (3)
B3 (male)	34	negative	• scapula alata<br>• horizontal axillary fold<br>• polyhill sign<br>• Beevor sign positive	• asymmetric proximal weakness of the upper limb and	-	519	NA	Histology: myopathic changes	FSHD	176 (3)

				mild proximal weakness of the lower limb<br>• foot drop palsy						
B4 (female)	75	negative	• myopathic face<br>• scapula alata<br>• horizontal axillary fold<br>• hyperlordosis	• facial weakness, asymmetric proximal weakness of the upper limb and mild weakness of the lower limb<br>• foot drop palsy<br>• axial weakness	-	~270-340	myopathic pattern	-	FSHD	80 (3)
B5 (female)	53	positive (mother of patient B6)	• myopathic face<br>• horizontal axillary fold	• facial weakness<br>• proximal weakness of the upper and lower limb	progressive bilateral sensorineural hearing loss since childhood	normal	NA	-	FSHD	132 (3.5)
B6 (male)	13	positive (son of mother B5)	• no definite clinical signs of FSHD	• mild proximal weakness of the upper limb and lower limb	progressive bilateral sensorineural hearing loss since childhood	normal	NA	-	myopathy	ND[d]
B7 (female)	34	negative	• myopathic face<br>• scapula alata<br>• polyhill sign<br>• Beevor sign<br>• hyperlordosis	• facial weakness<br>• asymmetric proximal weakness of the upper and lower limb<br>• foot drop palsy	-	~ 300–1000	NA	NA	FSHD	179 (5)
FSHD2 with variant of unknown significance in SMCHD1										
B8 (male)	67	positive (twin brother of patient B9)	• scapula alata	• mild facial weakness<br>• proximal weakness of the upper and lower limb<br>• foot drop palsy	-	NA	NA	-	FSHD	89 (3)
B9 (male)	67	positive (twin brother of patient B8)	• scapula alata	• proximal weakness of the upper and lower limb	-	NA	NA	-	FSHD	75 (2.5)

				• foot drop palsy						
B10 (male)	61	Positive (mother, grandmother)	• myopathic face<br>• scapula alata	• facial weakness<br>• asymmetric proximal weakness of the upper and lower limb<br>• foot drop palsy	-	normal	NA	-	FSHD	131 (4)

Abbreviations: CK: creatinine kinase; DD: differential diagnosis; NA: not available; ND: not determined; PSA: pathological spontaneous activity.

[a]Normal result: < 170 U/L.
[b]Not determined because patient was tested negative for FSHD.
[c]Not determined due to dual diagnosis phenotypically overlapping with FSHD.
[d]Not determined due to pattern of muscle involvement not sufficiently represented by the CSS.

Supplementary Table 4. Genetic and epigenetic findings in patients of Cohort 2. Results from haplotype analysis by Tsumagari et al.[7] and a KASP assay as previously described,[2] as well as methylation profile analysis (FSHD-MPA). Additionally, repeat lengths as determined by Optical genome mapping (OGM) and/or Southern blotting, results of NGS sequencing of epigenetic suppressor genes (*SMCHD1*, *DNMT3B*, *LRIF1*) and exome sequencing, as well as *SMCHD1*-MLPA analysis and OGM analysis of *SMCHD1*, *DNMT3B* and *LRIF* are given.

Patient (sex)	Haplotype analysis[a]	KASP assay	DUX4 assay methylation [%]		4qA assay methylation [%]		4qAL assay		OGM repeat analysis[b]	NGS sequencing of *SMCHD1, DNMT3B, LRIF1*	OGM analysis of *SMCHD1, DNMT3B, LRIF1*	*SMCHD1*-MLPA	NGS exome *or* genome sequencing
FSHD with 11 RU in absence of global hypomethylation (FSHD$_{11\ RU}$)													
D1 (female)	4qA161/4qB163	+	43.5	(+)	/	/	51.4	+	9 RU 4qB/11 RU 4qA<br>26 RU 10qA/34 RU 10qA;<br>11 RU Southern Blot	negative	ND	ND	negative
FSHD2 without causative variant in SMCHD1, LRIF1, DNMT3B (FSHD2$_{no\ variant}$)													
E1 (female)	4qA161	+	40.2	+	31.5	+	/	/	16 RU 4qA/42 RU 4qA<br>5 RU 10qA/20 RU 10qA	negative	negative	negative	negative
E2 (male)	4qA161/4qB163	+	32.7	+	27.0	+	53.1	(+)	16 RU 4qA/19 RU 4qA<br>9 RU 10qA/19 RU 10qB	negative	negative	negative	negative
E3 (male)	4qA161/4qA161	+	39.1	+	41.6	(+)	/	/	12 RU 4qA/15 RU 4qA<br>8 RU 10qA/18 RU 10qA	negative	negative	negative	negative
E4 (male)	4qA161	+	40.2	+	24.7	+	/	/	14 RU 4qA/21 RU 4qB<br>7 RU 10qA/20 RU 10qA	negative	negative	negative	*DOK7*-associated CMS:<br>NM_173660.5:<br>1) c.1124_1127dup, p.(Ala378Serfs*30), heterozygous, pathogenic (PVS1_str, PS3_sup, PM3_str, PP1)<br>2) c.54+25_55-38del, p.(Trp19Valfs*24), heterozygote, likely pathogenic (PS4_mod, PM3, PM2_sup, PP3)
E5 (male)	4qA161	+	36.8	+	37.7	(+)	/	/	12 RU 4qA/16 RU 4qA<br>10 RU 10qA/19 RU 10qA	negative	negative	negative	negative
E6 (female)	4qA161	+	37.6	+	26.5	+	/	/	13 RU (Southern blot)	negative	negative	negative	negative
Non-pathogenic complex 4qA166 repeat array													
F1 (female)	4qA161	+	51.9	-	60.4[c]	-	/	/	***OGM:***<br>7 RU 4qA/<br>43 RU 4qA; 12 RU 10qA/<br>24 RU 10qA<br>***Southern blot*** (p13E11 probe):	negative	ND	ND	negative

									EcoRI: 31 kb, 48 kb, >48 kb fragments; *EcoRI* + *BlnI*: no fragment <48 kb; *XapI*: 4-5 kb and 43 kb				
Complex genetic findings in light of an Asian population background (FSHD$_{South\ Asian\ population}$)													
G1 (male)	4qA161/4qB163	+	36.9	+	17.7	+	/	/	7 RU 4qA/14 RU 4qB<br>6 RU 10qA/19 RU 10qA	*DNMT3B:* NM_006892.4: c.2361A>C, p.(Gln787His), heterozygous, VUS (PM2_sup)	ND	ND	negative
G2 (female)	4qA161	+	50.5	-	49.5	-	/	/	9 RU 4qA/24 RU 4qA<br>13 RU 10qA/21 RU 10qA	*DNMT3B, SMCHD1, LRIF1:* negative	ND	ND	negative
G3 (female)	4qA161/4qB163	+	41.4	(+)	24.5	+	/	/	9 RU 4qA/14 RU 4qB<br>6 RU 10qA/21 RU 10qA	*DNMT3B:* NM_006892.4: c.2361A>C, p.(Gln787His), heterozygous, VUS (PM2_sup)	ND	ND	negative
FSHD2 with (likely) pathogenic variant in SMCHD1 (FSHD2$_{SMCHD1}$)													
B2 (male)	4qA161	+	28.0	+	13.8	+	/	/	21 RU 4qA/22 RU 4qB<br>7 RU 10qA/12 RU 10qA	*SMCHD1*: NM_015295.3: c.2700+1_2700 +3dup p.?, heterozygous, pathogenic (PVS1, PS3_mod, PM2_sup)	ND	ND	negative
B3 (male)	4qA161/4qB163	+	35.0	+	10.3	+	/	/	13 RU 4qA/16RU 4qA<br>23 RU 10qA/41 RU 10qA	*SMCHD1*: NM_015295.3: c.2701-2A>T p.?, heterozygous, likely pathogenic (PVS1, PM2_sup)	ND	ND	negative
B4 (female)	4qA161	+	30.6	+	19.7	+	/	/	23 RU 4qA/25 RU 4qB<br>6 RU 10qA/20 RU 10qA	*SMCHD1*: NM_015295.3: deletion Exon 4 und 5, heterozygous,	ND	exon 4, 5 deletion confirmed	negative

										pathogenic (PVS1, PS3_sup, PM2_sup)			
B5 (female)	4qA161/4qB163	+	39.3	+	26.3	+	/	/	27 RU 4qA/16 RU 4qB 19 RU 10qA/21 RU 10qA	*SMCHD1*: NM_015295.3: c.262+1G>A, p.?, heterozygous, likely pathogenic (PVS1, PM2_sup)	ND	ND	*EYA4*: NM_004100.5: c.494del, p. Gly165Alafs*33, heterozygous, likely pathogenic (PVS1, PM2_sup)
B6 (male)	4qA161	+	43.6	(+)	25.2	+	/	/	27 RU 4qA/37 RU 4qB 19 RU 10qA/43 RU 10qA	*SMCHD1*: NM_015295.3: c.262+1G>A, p.?, heterozygous, likely pathogenic (PVS1, PM2_sup)	ND	ND	*EYA4*: NM_004100.5: c.494del, p. Gly165Alafs*33, heterozygous, likely pathogenic (PVS1, PM2_sup)
B7 (female)	4qA161/4qA161	+	30.6	+	17.0	+	/	/	12 RU 4qA/24 RU 4qA 1 RU 10qA/27 RU 10qA	*SMCHD1*: NM_015295.3: c.1520del (p.Asn507Ilefs*20) heterozygous, pathogenic (PVS1, PM2_sup, PS3_sup)	ND		negative
FSHD2 with variant of unknown significance in SMCHD1													
B8 (male)	4qA161	+	38.4	+	20.9	+	62.5	+	22 RU 4qA/59 RU 4qA 9 RU 10qA/15 RU 10qA	*SMCHD1*: NM_015295.2: c.5306G>A (p.Gly1769Asp), heterozygous, VUS, (PM2_sup, PP3, PP2)	ND	ND	negative
B9 (male)	4qA161	+	28.5	+	29.0	+	48.8	+	19 RU 4qA/22 RU 4qA 9 RU 10qA/11 RU 10qA	*SMCHD1*: NM_015295.2: c.5306G>A (p.Gly1769Asp), heterozygous,	ND	ND	negative

B10 (male)	4qA161/4qB163	+	39.9	+	10.1	+	/	/	11 RU 4qA/15 RU 4qB<br>7 RU 10qA/22 RU 10qA	VUS (PM2_sup, PP3, PP2)<br>*SMCHD1:* NM_015295.3: c.5548-4A>G p.?, heterozygous, VUS (PS3_sup, PM2_sup, PP3)	ND	ND	negative

Abbreviations: ND: not determined, CMS: congenital myasthenic syndrome, KASP: Kompetitive allele specific PCR, MLPA: multiplex ligation-dependent probe amplification; NGS: next-generation sequencing, OGM: Optical Genome Mapping.

[a] Based on single nucleotide polymorphisms (SNPs) in the region proximal to the 4q35 D4Z4 repeat array following the protocol of Tsumagari *et al.* SNP combinations of 4qA166/4qB168/10qA166 are identical. Therefore, in some cases it is not possible to differentiate whether patients are homozygous for 4qA161 and carry the 10qA166 haplotype or heterozygous for 4qA161 and carry the 4qB168 haplotype at the other allele. For these patients only one haplotype (4qA161) is given in the Table.

[b] OGM cannot distinguish the 4qA and 4qAL haplotype. In both cases, the result is 4qA.

[c] 4qA166 allele is not amplified by FSHD-MPA by primer design. Distal methylation only represents the other permissive allele.

Supplementary Table 5. Cut-off values for methylation levels (positive, negative, and inconclusive) in the 4qA, 4qAL and DUX4 assay of FSHD-MPA.

Assay	positive	inconclusive	negative
**4qA**	< 0.347	0.347–0.442	> 0.442
**4qAL**	< 0.526	0.526–0.620	> 0.620
**DUX4**	< 0.404	0.403–0.456	> 0.456

Supplementary Table 6. Sequence of CRISPR/Cas9 RNAs (crRNAs) used for the enrichment of the 4q35 repeat array in the ONT long-read sequencing assay.

crRNA	location	Sequence 5'→3'
**G1**	proximal	CCTATTAAACGTCACGGACA
**G2**	distal	AAATCTTCTATAGGATCCAC

Supplementary Table 7. Distal and proximal anchor sequence used in the bioinformatics workflow.

location	Sequence 5'→3'
**proximal anchor**	ACAAGGCCAGAGTTTGAATATACTGTGGTCATCTCTGCTCCAGTGCAAACTGTTTCCAGAAAGCCTACTTC<br>TATTTTCCTTGCTGTAACAGAGGAACATTTCCTGTCTTATGTTTATTCTACTCTGCAATCCCCTAAGGCTTTT<br>TCTCTCCCTCCCAGAATCTTAAAGTGCATTCGAACTCACAGGCAAAATCCTCCCAGAATCTTGTGAGAACA<br>TAAATGATCTGACTAGTTTGGCATTGCTTTTGGGGATCTGGGAAAATCTGTGCACACTTCTGGAGACCCTT<br>GTCATGCCATTTTTTATAAATCTATTGTGCCTCAAGTCAGAAGTGTGTGAGGGGAGATGGGGAGACATTGG<br>GATGCGCGCGCCTGGGGCTCTCCCACAGGGGGCTTTCG
**distal anchor**	CCGGGCCCCTGCAGCCTCCCAGCTGCCAGCGCGGAGCTCCTGGCGGTCAAAAGCATACCTCTGTCTGTC<br>TTTGCCCGCTTCCTGGCTAGACCTGCGCGCAGTGCGCACCCCGGCTGACGTGCAAGGGAGCTCGCTGGC<br>CTCTCTGTGCCCTTGTTCTTCCGTGAAATTCTGGCTGAATGTCTCCCCCCACCTTCCGACGCTGTCTAGGC<br>AAACCTGGATTAGAGTTACATCTCCTGGATGATTAGTTCAGAGATATATTAAAATGCCCCCTCCCTGTG

Supplementary Table 8. Quality parameters for all ONT LRS runs performed in this study including flow cell type, total and on-target bases sequenced per run, mean read lengths, N50 values and mean read quality.

		total				on-target			
**patient**	**flow cell**	**total bases [Mb]**	**mean read length [bp]**	**N50 [bp]**	**mean read quality**	**total bases [Mb]**	**mean read length [bp]**	**N50 [bp]**	**mean read quality**
A1	R9	840.8	6829.6	14083	13.9	10.33	11819.6	15089	12.1
A2	R9	607.4	5127.8	12343	14.1	5.7	9426.3	24313	12.6
A3	R9	1233.6	10010.3	17525	14.1	13	15111.6	21466	12.3
A4	R9	3682.2	13391.3	20740	14.1	27.5	16612.6	18224	12.2
A5	R9	718.5	5061.4	13217	14.2	4.7	10978.1	21187	12.5
A6	R9	665.9	6814.7	13102	14.3	2.2	11304.8	21271	12.5
A7	R9	1212.3	14901.8	21095	15.2	4.8	18259	21225	13
A8	R9	2433.3	14522.2	26556	14.6	8.4	18609.8	31254	12.5
A9	R9	722	9490.7	14507	14.4	4.3	10313.9	13223	12.6
A10	R9	3010.3	11198.7	23966	15	8.1	15691.6	36136	13.1
A11	R9	591.9	9867	15.121	13.6	6.4	9369.9	8516	12
A12	R10	1814.6	5061.6	14964	17.5	5.2	12175.5	18333	17.9
B1	R9	565.4	7858	14466	15.2	2.8	14268.8	28155	13
C1	R9	2785.4	19421.4	34361	14.5	27.7	19605.5	23073	12.5
E2	R10	2196.7	14436.1	23273	18.5	6.2	21450.5	31614	17.3
F1	R9	930.3	8481.9	15921	13.8	9.7	14816.1	24675	12.1
G1	R9	650.3	4566.5	11137	15	2.8	9438.7	17210	13.1

Supplementary Table 9. Overall and on-target read counts for all ONT LRS runs performed. Overall reads generated per run, total number of on-target reads (4q35 and 10q26), and the proportion of on-target reads covering the distal pLAM sequence are provided. The 4q35-associated reads section reports the total number of reads covering the distal pLAM sequence, including the subset of full-length reads, and further stratified by unambiguous assignment to allele 1 or allele 2.

			on-target reads		4q35-associated reads					
**Patient**	**flow cell**	**Total reads**	**total**	**pLAM [%]**	**pLAM**	**all (full length reads)**	**allele 1**	**allele 1 (full length reads)**	**allele 2**	**allele 2 (full length reads)**
A1	R9	121252	881	54,94%	205	107	107	107	7	0
A2	R9	117065	452	46,24%	149	57	57	57	2	0
A3	R9	119843	818	56,48%	336	149	237	149	45 (AL), 52 (B)	0 (AL), 0 (B)
A4	R9	264289	1654	57,13%	612	453	453	453	0	0
A5	R9	140451	437	43,71%	140	69	69	69	4	0
A6	R9	96457	197	43,65%	46	13	13	13	1	0
A7	R9	79604	271	50,18%	88	59	59	59	11	0
A8	R9	161183	465	45,16%	120	85	85	85	24	0
A9	R9	74233	430	47,67%	146	116	116	116	2	0
A10	R9	260326	534	55,62%	225	195	195	195	11	0
A11	R9	58687	701	54,21%	336	240	240	240	31	1
A12	R10	354415	433	50,81%	155	120	120	120	0	0
B1	R9	70216	198	42,42%	46	7	7	7	0	0
C1	R9	138021	1074	75,98%	386	299	320	283	66	16
E2	R10	146367	251	58,17%	69	12	36	6	33	6
F1	R9	108337	654	48,01%	202	91	91	91	5	0
G1	R9	139315	301	34,55%	47	15	15	15	0	0

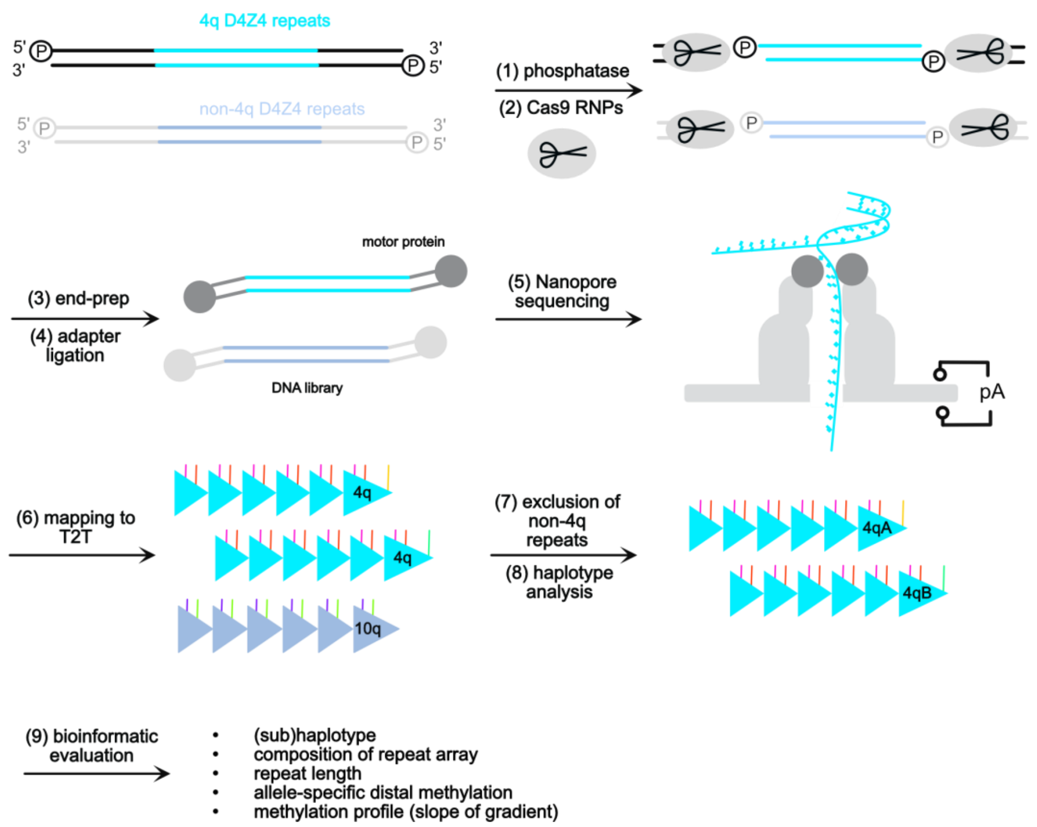

Supplementary Figure 3. ONT LRS approach for FSHD repeat analysis. Allele-specific analysis of 4q35 D4Z4 repeat arrays by ONT LRS. After (1) exclusion of all non-target fragments by dephosphorylation, (2) D4Z4 repeat arrays are enriched by CRISPR/Cas9 targeting the p13E11 region proximal and the pLAM region distal to the D4Z4 repeat array before (3, 4) library preparation. (5) Reads generated by ONT LRS are mapped to T2T and (7) D4Z4 repeat arrays from chromosome 4q35 are differentiated from those of homologous regions such as 10q26 based on SNPs being associated with specific restriction sides. Further bioinformatic analysis reveals additional parameter such as haplotype, subhaplotypes, repeat length, allele-specific distal methylation and methylation over the whole repeat arrays if accessible.

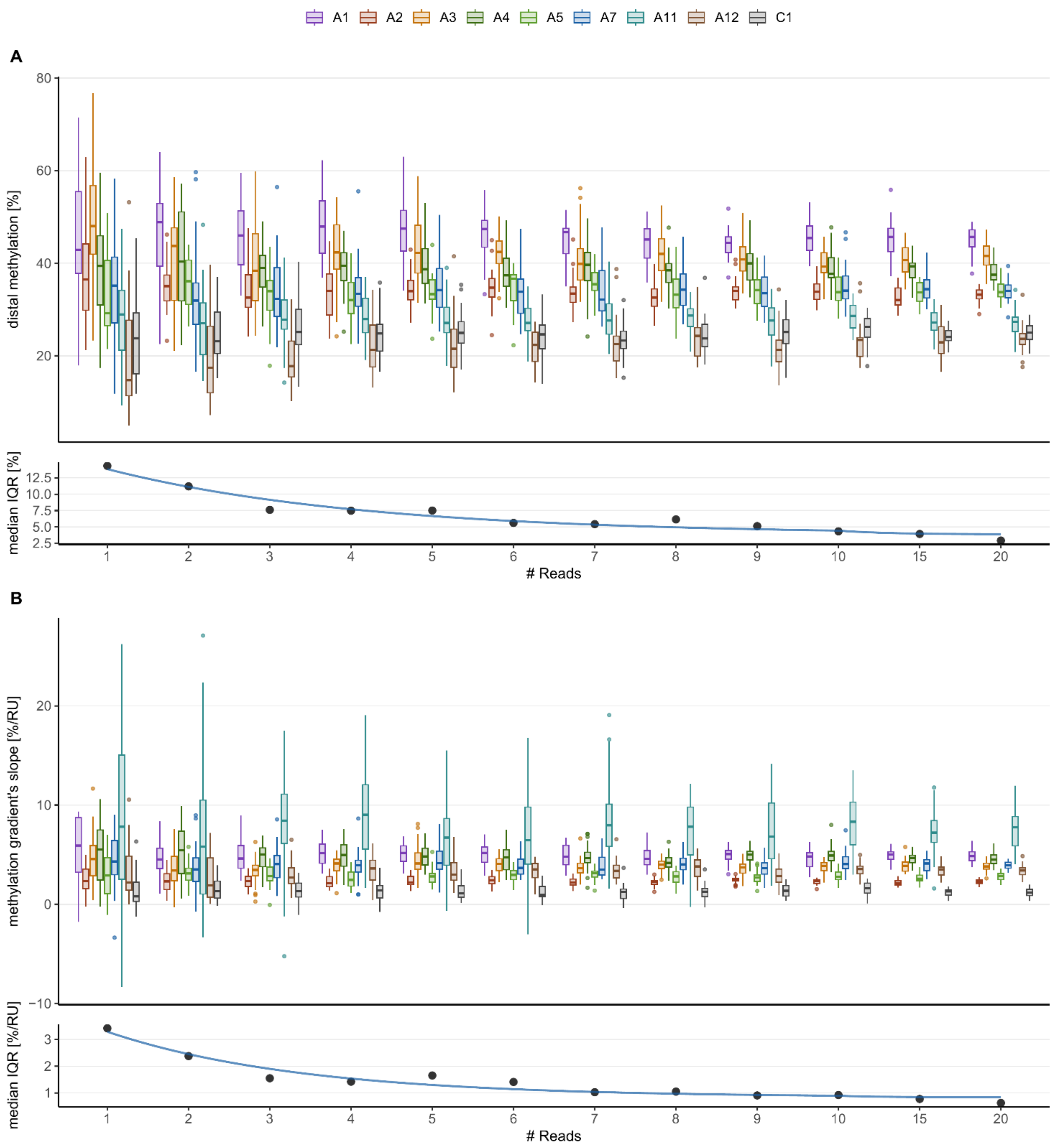

Supplementary Figure 4. Stability of distal methylation and methylation gradient's slope estimates across downsampling levels. Boxplots show the distribution of (A) distal methylation [%] and (B) methylation gradient's slope [%/RU] across 30 randomized downsamplings per read depth level for the contracted allele across nine samples (A1–A5, A7, A11, A12, C1). Lower panels display the median interquartile range (IQR) across all samples as a measure of dispersion, with the blue curve representing an exponential decay model ($f(x) = a \cdot e^{(-bx) + c}$) fitted to the data. Notably, sample A11 (2 RU) showed elevated variability in slope estimates; for samples with very short repeat arrays, a minimum of 15 reads per allele is recommended for slope analysis.

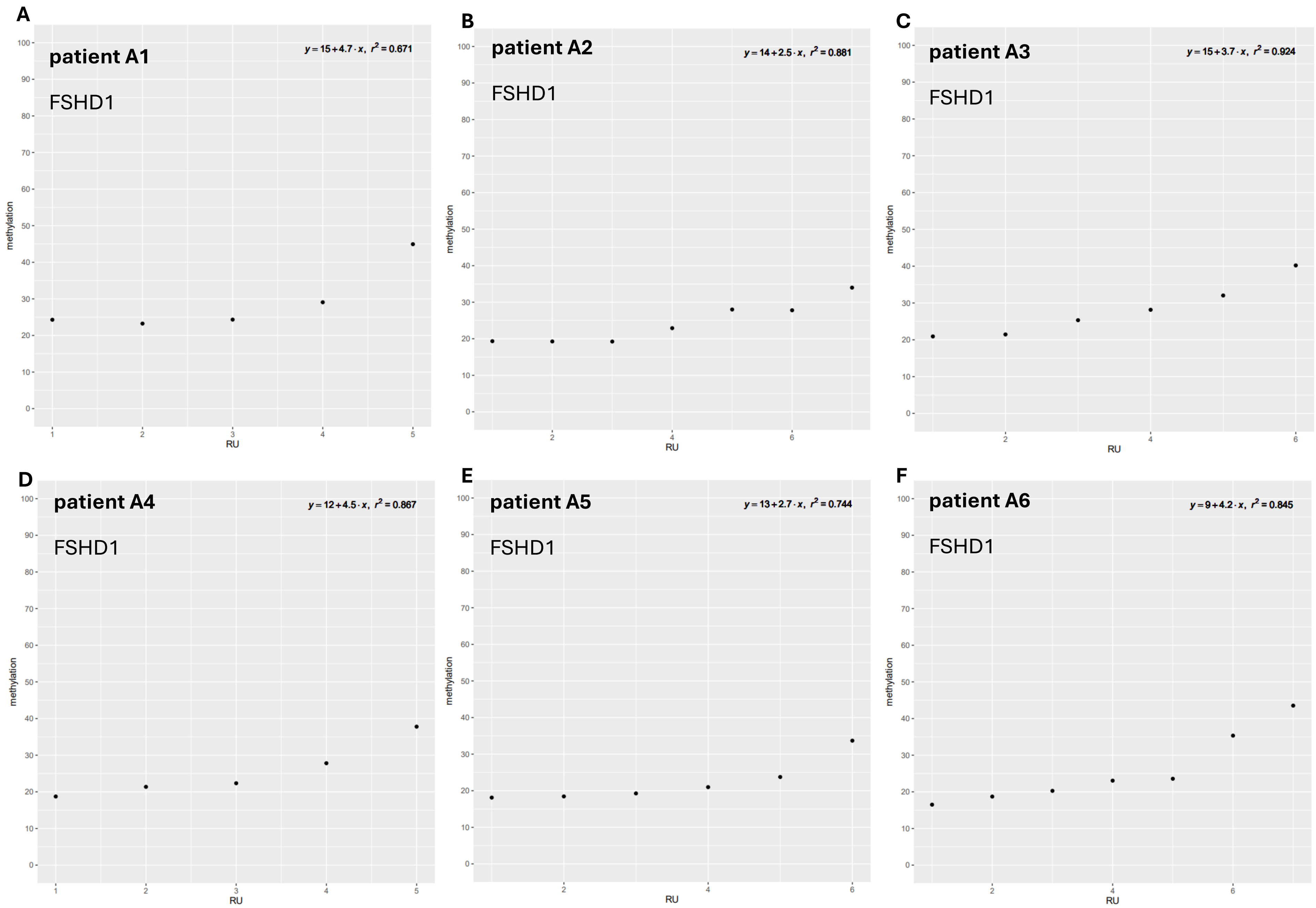
A
patient A1
FSHD1
y = 15 + 4.7 · x, r² = 0.671
B
patient A2
FSHD1
y = 14 + 2.5 · x, r² = 0.881
C
patient A3
FSHD1
y = 15 + 3.7 · x, r² = 0.924
D
patient A4
FSHD1
y = 12 + 4.5 · x, r² = 0.867
E
patient A5
FSHD1
y = 13 + 2.7 · x, r² = 0.744
F
patient A6
FSHD1
y = 9 + 4.2 · x, r² = 0.845
methylation
RU

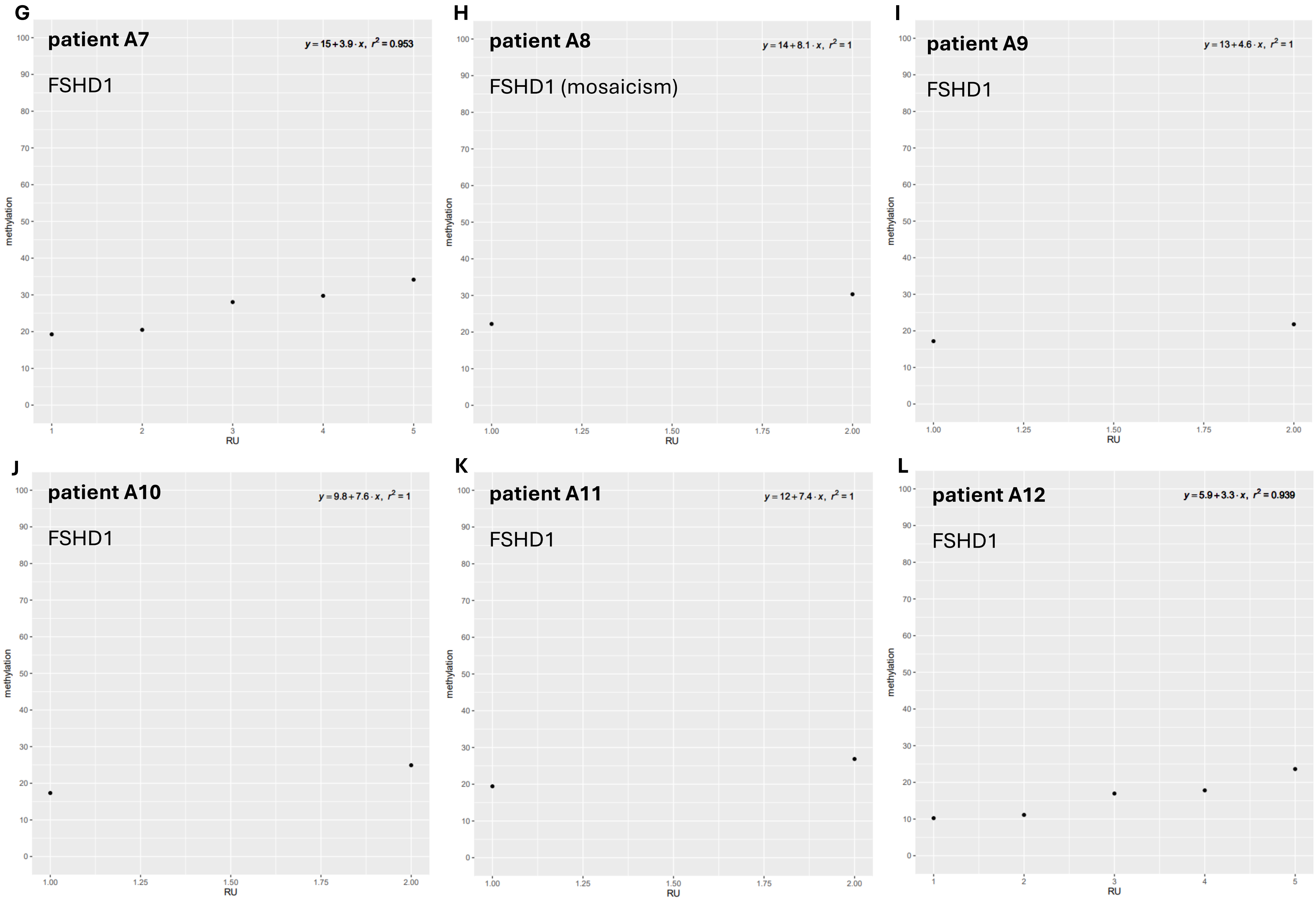
G
patient A7
FSHD1
$y = 15 + 3.9 \cdot x,\ r^2 = 0.953$
H
patient A8
FSHD1 (mosaicism)
$y = 14 + 8.1 \cdot x,\ r^2 = 1$
I
patient A9
FSHD1
$y = 13 + 4.6 \cdot x,\ r^2 = 1$
J
patient A10
FSHD1
$y = 9.8 + 7.6 \cdot x,\ r^2 = 1$
K
patient A11
FSHD1
$y = 12 + 7.4 \cdot x,\ r^2 = 1$
L
patient A12
FSHD1
$y = 5.9 + 3.3 \cdot x,\ r^2 = 0.939$
methylation
RU

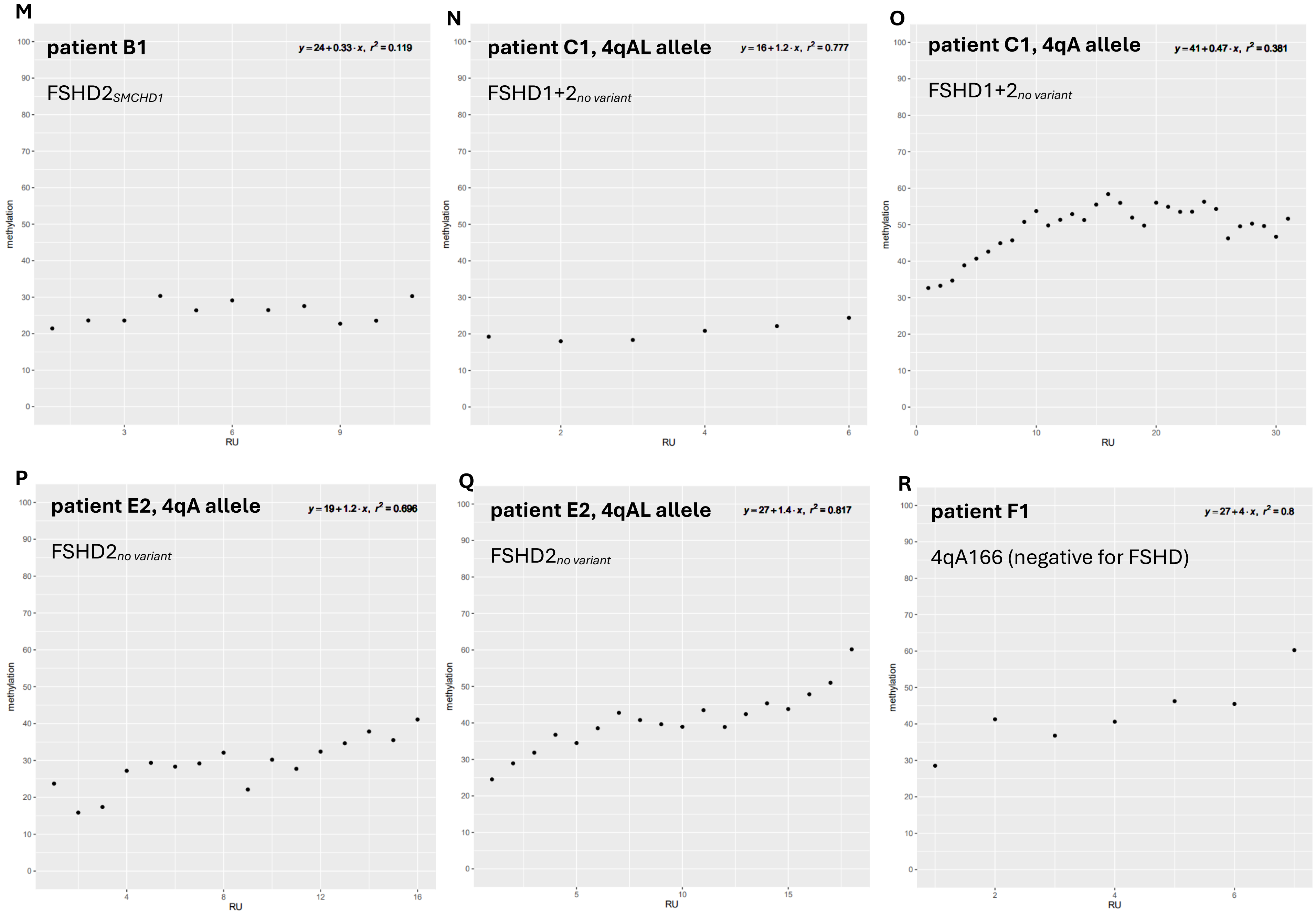
M
patient B1
FSHD2SMCHD1
y = 24 + 0.33 · x, r2 = 0.119
N
patient C1, 4qAL allele
FSHD1+2no variant
y = 16 + 1.2 · x, r2 = 0.777
O
patient C1, 4qA allele
FSHD1+2no variant
y = 41 + 0.47 · x, r2 = 0.381
P
patient E2, 4qA allele
FSHD2no variant
y = 19 + 1.2 · x, r2 = 0.696
Q
patient E2, 4qAL allele
FSHD2no variant
y = 27 + 1.4 · x, r2 = 0.817
R
patient F1
4qA166 (negative for FSHD)
y = 27 + 4 · x, r2 = 0.8
methylation
RU

S

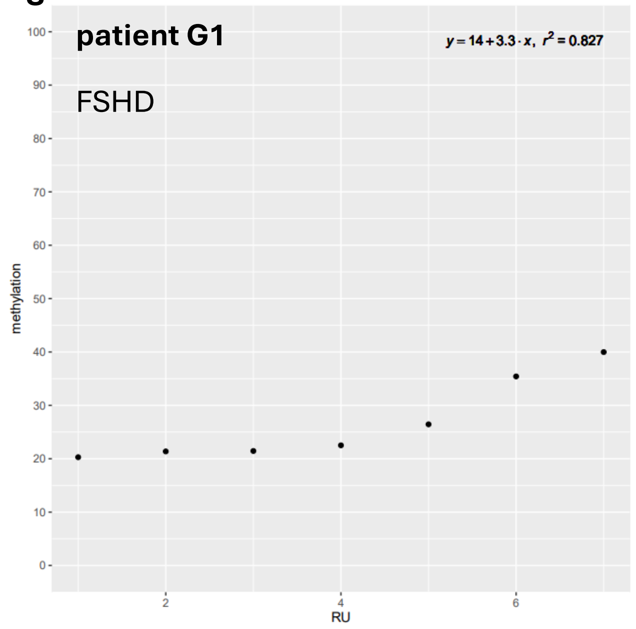

Supplementary Figure 5. Methylation profile of the shorter repeat array of all patients of Cohort 1 (n = 14, patients A1–A12, B1, C1) and three patients of Cohort 2 (n = 3 analyzed by LRS)- different groups of patients. Per plot the shorter 4q35 repeat array from one patient is represented. For patients C1 and E2 also the second 4q35 repeat array is shown. One dot represents the average methylation of one repeat unit across all reads obtained. Repeat units are numbered from the proximal to the distal end. Linear function and the square of Pearson's coefficient derived from linear regression analysis of the average CpG methylation per repeat unit (y-axis) and repeat length (x-axis) are given in the right upper corner.

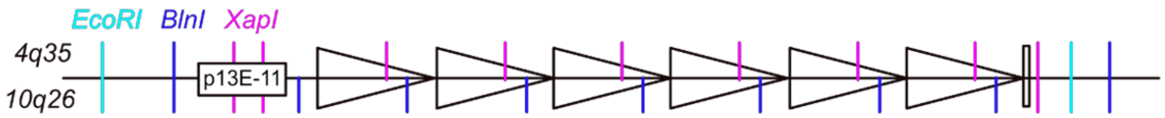

Supplementary Figure 6. Different restriction sites at 4q35 and 10q26 repeat arrays. Restriction enzymes *EcoRI*, *BlnI* and *XapI* allow for the differentiation of DNA fragments originating from chromosome 4 (resistant to *BlnI*, sensitive to *XapI* = B^-X^+) and 10 (sensitive to *BlnI*, resistant to *XapI* = B^+X^-).

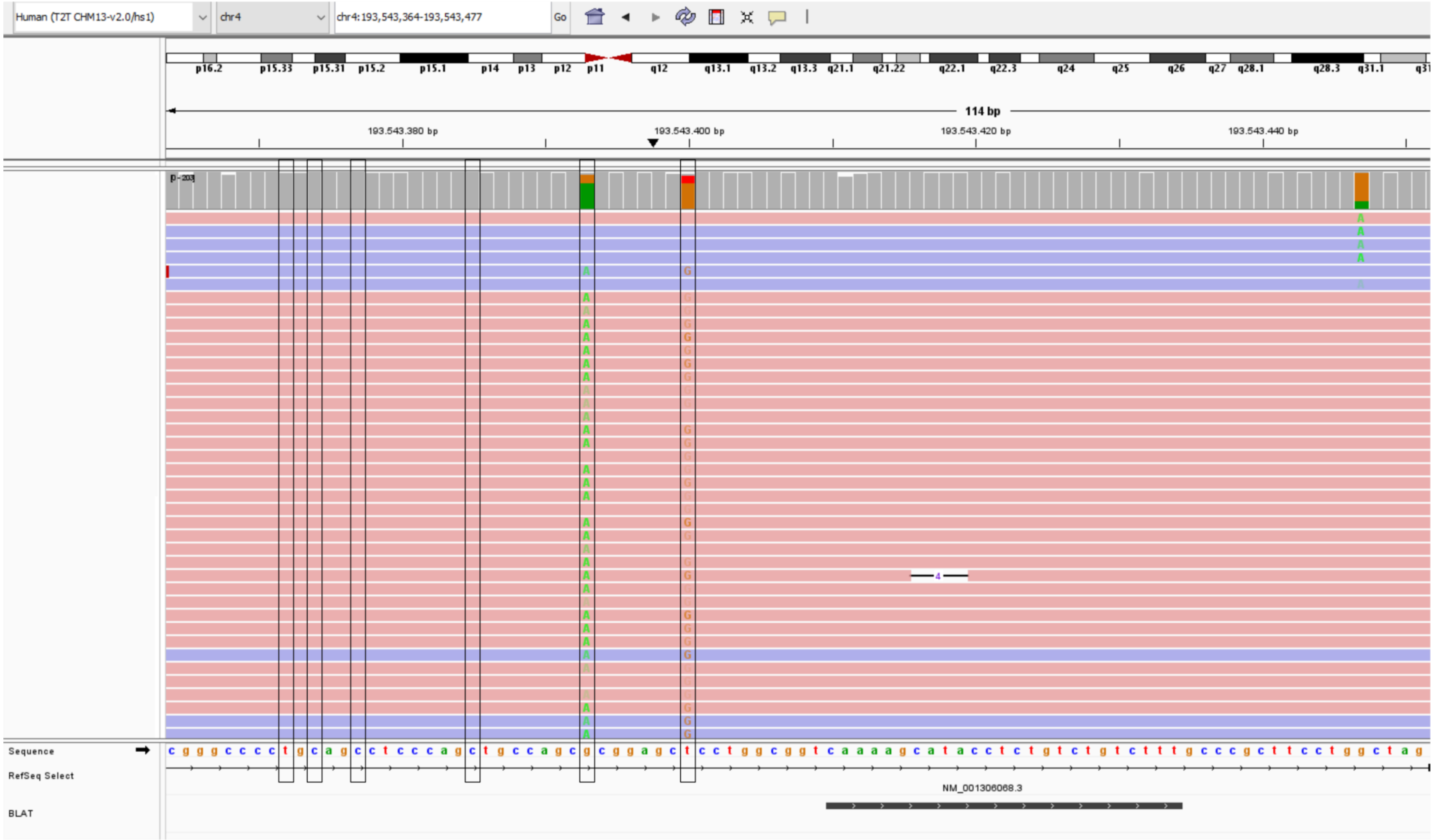

Supplementary Figure 7. Combination of single nucleotide polymorphisms at the 7 RU 4q35 repeat array of patient F1 within the region in between the distal repeat unit and the pLAM region defining the 4qC166H haplotype according to [8].

Supplementary Table 10. Comparison of different methods to diagnose FSHD.

Method	Material	Turnaround time	Costs	Diagnostic parameter	Detection of	Diagnostic gaps and comments
Standard Southern blot (p13E11 probe, linear gel electrophoresis)	high-molecular weight DANN (high amounts ~30–50 µg)	weeks	+	repeat length up to 48 kb (~ 13 RU)	FSHD1	- unknown penetrance of intermediate repeat arrays (8–10 RU in the European/North American population)<br>- missing repeat length thresholds for several populations<br>- no detection of FSHD2 possible<br>- haplotype is not routinely determined and requires additional probes<br>- separate haplotyping does not allow to determine the configuration (*cis/trans*) to a repeat contraction<br>- Southern blot can indicate presence of complex alleles without being able to resolve their structure<br>- medium-level somatic mosaicism can be detected, but not differentiated from non-mosaic repeat contractions (can be overcome by pulse field gel electrophoresis)
Optical Genome Mapping	ultra-high-molecular weight DNA (~ 1 µg)	days	++	repeat length of all 4q and 10q repeat arrays independent of their size and their haplotype	FSHD1	- unknown penetrance of intermediate repeat arrays (8–10 RU in the European/North American population)<br>- missing repeat length thresholds for several populations<br>- no detection of FSHD2 possible<br>- complex repeat arrays (e.g. *in cis* duplications, hybride alleles, 4q10q rearrangements) can usually not be resolved (such patients appear homozygous for one 4qA allele in the automated workflow)<br>- alterations within the repeat array are missed, e.g. associated with the 4qA166 haplotype<br>- detection of somatic mosaicism possible depending on coverage
NGS sequencing of FSHD2 genes (*SMCHD1*, *LRIF1*, *DNMT3B*)	standard DNA (~100–500 ng)	days–weeks	++	pathogenic variants in epigenetic suppressor genes *SMCHD1*, *LRIF1*, *DNMT3B* associated with FSHD2	possible FSHD2	- no detection of FSHD1<br>- presence of pathogenic variant in epigenetic suppressor genes does not confirm FSHD2 (additional haplotype analysis is required)<br>- CNVs, SVs and deep intronic variants might be missed
Bisulfite sequencing based on methylation assays[1–4,9]	standard DNA (~ 1 µg)	days–weeks	+ (Sanger)/ ++ (nGS)	- distal methylation within the last 4q35 RU of permissive alleles<br>- global methylation (average methylation of all 4q and 10q RUs)	FSHD1 and FSHD2	- can detect FSHD1 and FSHD2 including $FSHD2_{no\ variant}$ patients<br>- configuration of permissive haplotype *in cis* to the hypomethylated alleles is confirmed<br>- estimation of penetrance of intermediate repeat sizes (8–10 RU in the European/North American population) and of alleles with 11 RU likely possible<br>- relevant amount of inconclusive results for FSHD1 patients being homozygous for the permissive haplotype, methods to compensate for this available[9,10]<br>- high risk of missing mosaic repeat contractions<br>- in principle capable to detect FSHD as result of a complex rearrangement without being able to reveal structure of the rearrangement<br>- not validated for predictive testing (but likely suitable)<br>- global methylation is biased by repeat length
Oxford Nanopore long-read sequencing	high-molecular weight DNA (~2–10 µg)	days	+++	- repeat length until a certain length<br>- allele-specific distal methylation<br>- global methylation as in BSS-assays<br>- methylation profile including slope of the gradient<br>- in principle parallel sequencing of epigenetic suppressor genes possible	FSHD1 and FSHD2	- potential of becoming the most precise and comprehensive diagnostics<br>- able to determine allele-specific distal methylation (major advantage to bisulfite based assays)<br>- able to determine slope of the methylation gradient as indicator for epigenetic modifier not being biased by repeat length in contrast to global methylation<br>- in principle able to decipher complex rearrangements<br>- ability to detect mosaic repeat contractions depends on coverage<br><br>- currently no validated diagnostic workflow available

References

1. Jones TI, Yan C, Sapp PC, et al. Identifying diagnostic DNA methylation profiles for facioscapulohumeral muscular dystrophy in blood and saliva using bisulfite sequencing. *Clin Epigenetics*. 2014;6(1):23.

2. Erdmann H, Scharf F, Gehling S, et al. Methylation of the 4q35 D4Z4 repeat defines disease status in facioscapulohumeral muscular dystrophy. *Brain*. 2023;146(4):1388-1402.

3. Calandra P, Cascino I, Lemmers RJLF, et al. Allele-specific DNA hypomethylation characterises FSHD1 and FSHD2. *J Med Genet*. 2016;53(5):348-355.

4. Hartweck LM, Anderson LJ, Lemmers RJ, et al. A focal domain of extreme demethylation within D4Z4 in FSHD2. *Neurology*. 2013;80(4):392.

5. van Overveld PGM, Lemmers RJFL, Sandkuijl LA, et al. Hypomethylation of D4Z4 in 4q-linked and non-4q-linked facioscapulohumeral muscular dystrophy. *Nat Genet*. 2003;35(4):315-317.

6. Strafella C, Caputo V, Bortolani S, et al. Whole exome sequencing highlights rare variants in *CTCF*, *DNMT1*, *DNMT3A*, *EZH2* and *SUV39H1* as associated with FSHD. *Front Genet*. 2023;14:1235589.

7. Tsumagari K, Chen D, Hackman JR, Bossler AD, Ehrlich M. FSH dystrophy and a subtelomeric 4q haplotype: a new assay and associations with disease. *J Med Genet*. 2010;47(11):745.

8. Lemmers RJLF, van der Vliet PJ, van der Gaag KJ, et al. Worldwide Population Analysis of the 4q and 10q Subtelomeres Identifies Only Four Discrete Interchromosomal Sequence Transfers in Human Evolution. *Am J Hum Genet*. 2010;86(3):364-377.

9. Strafella C, Megalizzi D, Trastulli G, et al. Integrating D4Z4 methylation analysis into clinical practice: improvement of FSHD molecular diagnosis through distinct thresholds for 4qA/4qA and 4qA/4qB patients. *Clin Epigenetics*. 2024;16(1):148.

10. Xia X, Cheng N, Liu Y, et al. 4qA D4Z4 Methylation Test as a Valuable Complement for Differential Diagnosis in Patients with a Facioscapulohumeral Muscular Dystrophy–Like Phenotype. *J Mol Diagn*. 2025;27(5):405-418.
